# Anti-Obesity Potential of Ponciri Fructus: Effects of Extracts, Fractions and Compounds on Adipogenesis in 3T3-L1 Preadipocytes

**DOI:** 10.3390/molecules27030676

**Published:** 2022-01-20

**Authors:** Gopal Lamichhane, Prakash Raj Pandeya, Ramakanta Lamichhane, Su-jin Rhee, Hari Prasad Devkota, Hyun-Ju Jung

**Affiliations:** 1 Department of Oriental Pharmacy and Wonkwang-Oriental Medicines Research Institute, Wonkwang University, Iksan 570-749, Korea; lamichhanegopal1@gmail.com (G.L.); pandeya.praj@gmail.com (P.R.P.); clickrama@hotmail.com (R.L.); 2 Department of Pharmacy, College of Pharmacy, Wonkwang University, Iksan 570-749, Korea; rheesj05@wku.ac.kr; 3 Graduate School of Pharmaceutical Sciences, Kumamoto University, 5-1 Oe-honmachi, Chuo ku, Kumamoto 862-0973, Japan; devkotah@kumamoto-u.ac.jp

**Keywords:** Ponciri Fructus, anti-adipogenic activity, phellopterin, poncirin, oxypeucedanin, 3T3-L1 preadipocyte

## Abstract

Background: Ponciri Fructus, a crude drug consisting of the dried immature fruits of *Poncirus trifoliata* (L.) Raf., is a popular folk medicine used for the treatment of allergy and gastrointestinal disorders in Korea and China. In this study, the anti-adipogenic activity of extracts and isolated compounds were evaluated using 3T3-L1 preadipocytes. Methods: Dried immature fruits were extracted and fractionated into *n*-hexane, ethyl acetate (EtOAc), *n*-butanol and water-soluble fractions. The ethanol extract and fractions were tested for anti-adipogenic activity in the 3T3-L1 cell line. The active fractions (*n*-hexane and EtOAc fractions) were further subjected to chromatographic techniques to isolate and identify active compounds. Furthermore, the isolated compounds were evaluated for their anti-adipogenic activity. Results: Altogether, seven compounds, including two flavonoids, one phytosteroid and four coumarin derivatives, were isolated. Ethanol extract, *n*-hexane fraction, EtOAc fraction and three isolated compounds (phellopterin, oxypeucedanin and poncirin) showed significant anti-adipogenic activity as observed by reduced lipid deposition in differentiated 3T3-L1 cells. Further, oxypeucedanin downregulated the key adipogenic markers, such as peroxisome proliferator-activated receptors proteins *γ* (PPAR-*γ*), sterol response element binding proteins-1 (SREBP-1), CCAAT/enhancer binding proteins-*α* (C/EBP-*α*), adipocyte-specific lipid binding proteins (FABP-4), adipocyte fatty acid binding proteins (aP2), lipoprotein lipase (LPL) and leptin. Conclusion: This study indicated that the ethanol extract, hexane fraction and ethyl acetate fraction of *P. trifoliata* fruits possess strong anti-adipogenic activity, containing the active compounds such as phellopterin, oxypeucedanin and poncirin. Further research is recommended to explore their efficacy and safety in animal and clinical models.

## 1. Introduction

Obesity is a clinical condition resulting from the disruption of homeostasis between food intake and energy expenditure causing a deposition of excess fat in adipocytes of the body [1]. A shift in the diets and lifestyle of people towards a reduced physical activity and consumption of processed food containing high amount of saturated fats and sugar has made the situation more vulnerable. As the obesity trend is increasing so fast, there is serious motivation to control both it and the resulting co-morbidities, such as diabetes mellitus, hypertension, dyslipidemia, coronary diseases and many cancers [2].

The uncontrolled differentiation of adipocytes in the white adipose tissues is a primary metabolic event that causes obesity [3]. The differentiation of fibroblast-like preadipocytes into insulin-responsive lipid-loaded mature adipocytes is called adipogenesis. Adipogenesis occurs in several stages under the influence of cascades of transcription factors [4]. Under high nutrition, an increase in the cyclic adenosine monophosphate (cAMPs) load in the cell triggers the release of cAMPs responsive elements binding proteins (CREB), which, in turn, activates adipogenesis signaling molecule CCAAT/enhancer binding proteins (C/EBP)-*β*, which further activate C/EBP-*α* and the peroxisome proliferator–activated receptors (PPAR)-*γ* cascade, forcing the cell to the terminal differentiation stage. This feedback loop of C/EBP-*α* and PPAR-*γ* induces the transcriptional activation of adipogenic genes, such as sterol response element binding proteins (SREBPs), fatty acid synthase (FAS), lipoprotein lipase (LPL), adipocyte-specific lipid binding proteins (FABP) and adipokines, which collectively results in the deposition of fatty acids and triglycerides in adipocytes [4,5,6,7]. Therefore, controlling the transcription of the adipogenic gene and proteins expression can be a good intervention to halt the process of obesity. Polyherbal formulation, such as 18KHT01, and isolated compounds from herbal resources (polygalin C and others), were found to be effective in controlling these adipogenic factors, indicating the potential of medicinal plants and isolated compounds in the management of adipogenesis and obesity [8,9,10,11].

The use of herbal medicine for the treatment of health ailments has dated back for centuries. Although the emergence of modern medicine has revolutionized the treatment methodology, folk medicine is still the major therapeutic approach in a large population of developing countries [12]. Herbal medicine has also served as a source of many important modern medicines and their intermediates [13]. Many plants have still not been completely studied for their chemical composition and bioactivity, indicating the tremendous potential source for the discovery of ideal new compounds and formulations for the treatment of diseases [13,14,15,16]. In this research work, we have selected Ponciri Fructus, a crude drug consisting of the dried immature fruits of *Poncirus trifoliata* (L.) Raf., a well-known Korean herbal medicine for studying anti-adipogenic activity in 3T3-L1 cells.

Ponciri Fructus fruit is widely used as an oriental medicine in Eastern Asia for the treatment of constipation, edema, dyspepsia, stomach ulcer, allergy, inflammation, hepatotoxicity and pulmonary diseases such as chest fullness, chest pain, bronchitis and sputum [17,18,19,20,21]. It is believed that, in a folk medicinal system, the fruit of this plant can break the stagnation of qi, thereby removing food retention, resolving phlegm and helping in the elimination of masses [19]. As the fruit has shown significant prokinetic activity, it serves as an ingredient in most of the over-the-counter medicine in South Korea for the treatment of gastrointestinal disorders [20]. This fruit regulates the motility of the small intestine, increasing the transit rate without altering gastric emptying in rat models. Besides, it was also found to stimulate the motility of the distal colon [21]. Several attempts had been made to isolate compounds from this fruit. Triterpenoid, coumarin and its derivatives, flavonoids, steroids and phenolic compounds have mainly been reported in the literature. Terpenoid, namely caryophyllene-*β*-oxide,25*α*, 21*β*-dimethylmelianodiol, 21*β*, 25-dimethylmelianodiol, 21*α*-methylmelianodiol, 21*β*–methylmelianodiol, hispidol-a-25-methyl ether and hispidol-b-25-methyl ether [22]; coumarins, such as methoxsalen [17], auraptene, isoimperatorin, isoschinilenol, bergapten, umbelliferone, scopoletin [22], poncimarin, oxypeucedanin methanolate, heraclenol 3”-methylester [23], limonin, imperatorin [24] and phellopterin [25]; and flavonoids, such as hesperidin, hesperidin methyl chalone [25], poncirin, poncirenin, naringin, (2*R*)-5-hydroxy-4′-methoxyflavanone-7-*O*-{*β*-glucopyranosyl-(1-2)-*β*-glucopyranoside [26], naringenin and neohesperidin [27], were isolated from this fruit. Other compounds such as avenalumic acid methyl ester [22], *β*-sitosterol [22], pancastatins A and B [28] were also reported in the fruit.

*Poncirus trifoliata* water extract has been reported to have anti-obesity, hypoglycemic and hypolipidemic activities in high-fat-diet-fed mice [29,30]. Similarly, a reduction inlow and very low-density lipoproteins was reported in hyperlipidemic rats [31]. The fruit extract was also found to decrease both the obesity-induced infiltration of macrophages and its resulting inflammations [32]. However, its effect on adipocyte differentiation and the possible mechanism is not studied. The objective of this study was to evaluate the antiadipogenic activity of the immature fruits of *P. trifoliata*. The ethanol extract and fractions of the fruits were used to evaluate the anti-adiopgenic activity in 3T3-L1 cells. The isolated compounds were also studied for identifying the molecular mechanism of anti-adipogenic activity in gene and protein levels.

## 2. Results

### 2.1. Structure Elucidation of Isolated Compounds

**Figure 1:**
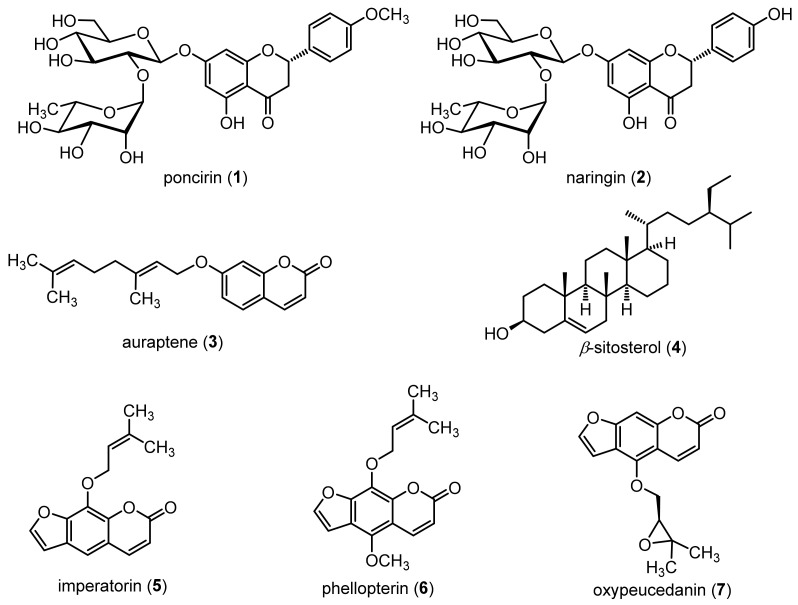
Structures of isolated compounds.

A total of 7 compounds were isolated from the ethanol extract (Figure 1). Compounds **1** and **2** were identified as poncirin and naringin, respectively. The conformation of these flavonoids was made based on exactly the same retention time and similar UV spectra as that of standard compounds (Merck, Darmstadt, Germany), as shown in Supplementary Figures S1 and S2 based on the ultra-performance liquid chromatography (UPLC) evaluation. The finding was further supported by a comparative study of reference proton (^1^H) and carbon-13 (^13^C) nuclear magnetic resonance (NMR) spectra (Figures S3 and S4) [33]. Compounds **3**, **5**, **6** and **7** were found to be coumarin derivatives, namely auraptene (**3**) [34], imperatorin (**5**) [35], phellopterin (**6**) [35] and oxypeucedanin (**7**) [35], respectively, based on a comparison of NMR spectra with literature values (Figures S5, S8, S11 and S13). The result is also supported by both infrared (IR) and mass spectra of compounds (Figures S6, S9, S10, S12 and S14–S16). Compound **4** was identified as *β*-sitosterol based on a comparison of the proton and carbon NMR spectra of the isolated compound with literature values (Figure S7) [36]. The obtained NMR spectra, IR and mass spectral data of all the compounds are presented in Supplementary Figures S1–S16.

### 2.2. UPLC Analysis of the Samples

**Figure 2:**
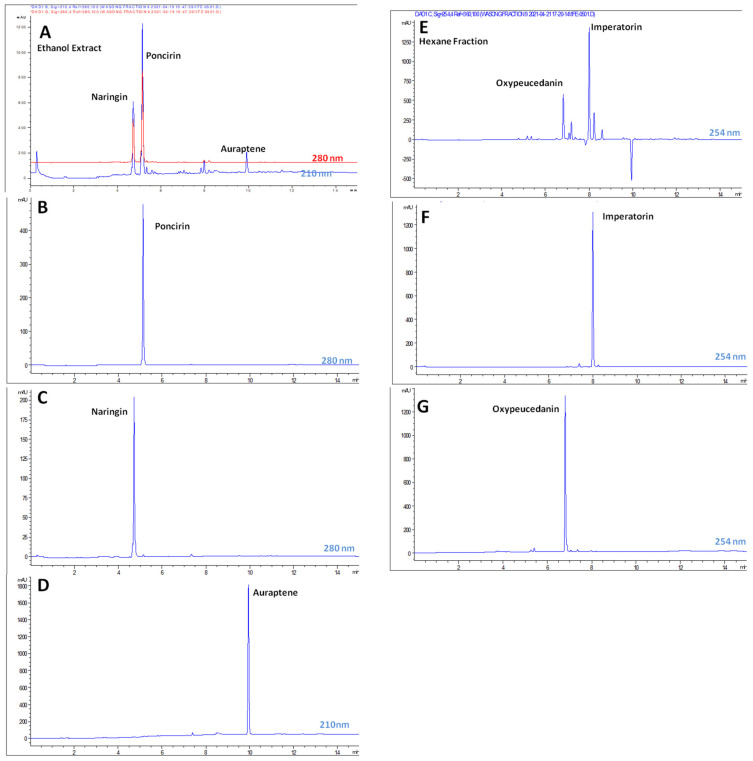
UPLC chromatograms of ethanol extract (**A**), poncirin (**B**), naringin (**C**), auraptene (**D**), hexane fraction (**E**), imperatorin (**F**) and oxypeucedanin (**G**).

An UPLC chromatogram of ethanol extract (Figure 2A), hexane fraction (Figure 2E) and isolated compounds was obtained through a developed methodology (Table S1). The amount of poncirin, naringin, auraptene, imperatorin and oxypeucedanin observed in the ethanol extract and hexane fractions was presented in Table 1. The results showed that poncirin, naringin and auraptene were major compounds present in the ethanol extract of the fruits.

### 2.3. Bioassay in 3T3-L1 Pre-Adipocyte

#### 2.3.1. Cell Viability Assay

**Figure 3:**
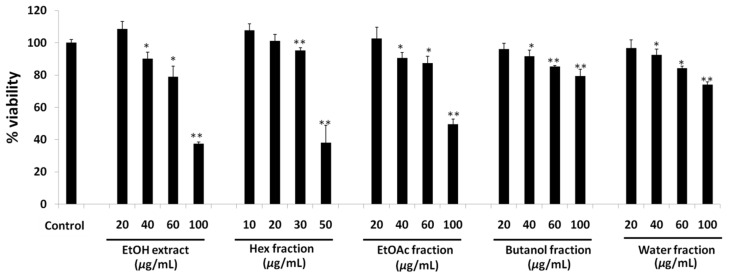
Cell viability of 3T3-L1 cell line on treatment with ethanol extracts, hexane fraction, ethyl acetate fraction, butanol fraction and water fraction of the Ponciri Fructus. Each set of data represents mean of triplicate experiment ± standard deviation. Significant difference between the groups was calculated using two-tailed Student’s *t*-test. * *p* ˂ 0.05 vs. control, ** *p* ˂ 0.01 vs. control are used to represent significant difference in cell viability of the extract sample compared to non-treated control group.

The cell viability of 3T3-L1 preadipocytes in treatment with ethanol extract, its fractions and its isolated compounds was evaluated using a 3-(4,5-dimethylthiazol-2-yl)-2,5-diphenyl tetrazolium bromide (MTT) assay. The results showed that the ethanol extract, as well as hexane, ethyl acetate, butanol and water fractions of ethanol extract, were nontoxic to 20 µg/mL in 3T3-L1 preadipocytes (Figure 3). An observation of the results showed that there was sharp increase in toxicity for the ethanol extract and hexane fraction with an increased dose, whereas a flat increase in toxicity was observed in ethyl acetate, butanol and water fraction, respectively. The obtained safe dose was used to evaluate the anti-adipogenic assay.

**Figure 4:**
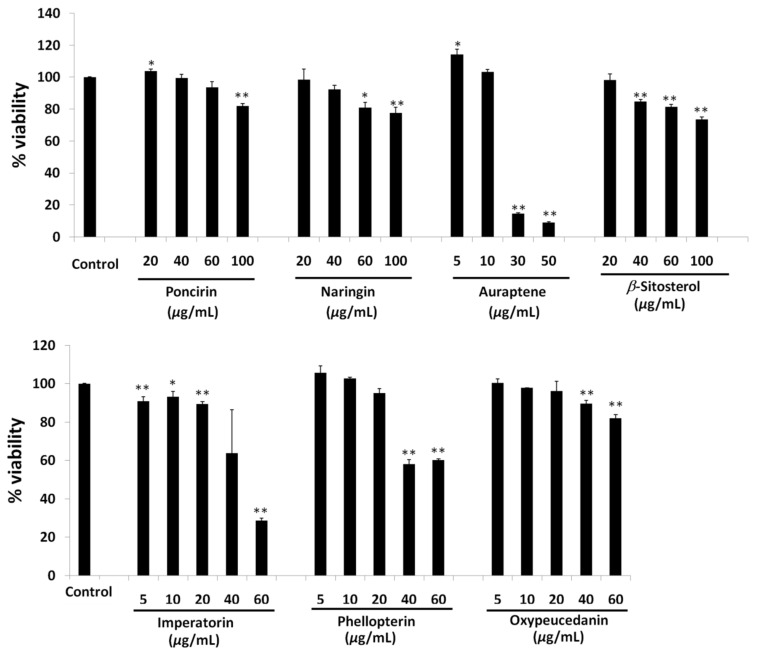
Cell viability of 3T3-L1 cell on treatment with poncirin, naringin, auraptene, *β*-sitosterol, imperatorin, phellopterin and oxypeucedanin isolated from Ponciri Fructus. Each set of data represents mean of triplicate experiment ± standard deviation. Significant difference between the groups was calculated using two-tailed Student’s *t*-test. * *p* ˂ 0.05 vs. control, ** *p* ˂ 0.01 vs. control are used to represent significant difference in toxicity of the extract sample compared to non-treated control group.

A cell viability assay of isolated compounds showed that poncirin and naringin were observed to be safe below 60 and 40 µg/mL concentrations, respectively (Figure 4). Auraptene, on the other hand, increased the cell viability significantly at a low dose of 5 µg/mL, whereas an abrupt increase in toxicity was observed at a 30 µg/mL concentration (Figure 4). *β*-Sitosterol was found to be safe below a 20 µg/mL dose (Figure 4). Imperatorin showed a 93.35 ± 2.62% cell viability at 10 µg/mL concentrations, whereas a cell viability of 89.5±1.37% was observed at a 20 µg/mL dose (Figure 4). This is significantly lower compared to the control group. An abrupt reduction in cell viability was observed above a 20 µg/mL concentration of imperatorin. Phellopterin and oxypeucedanin were found to be safe up to a 20 µg/mL dose for treatment in 3T3-L1 cells (Figure 4).

#### 2.3.2. Oil Red O (ORO)Assay

**Figure 5:**
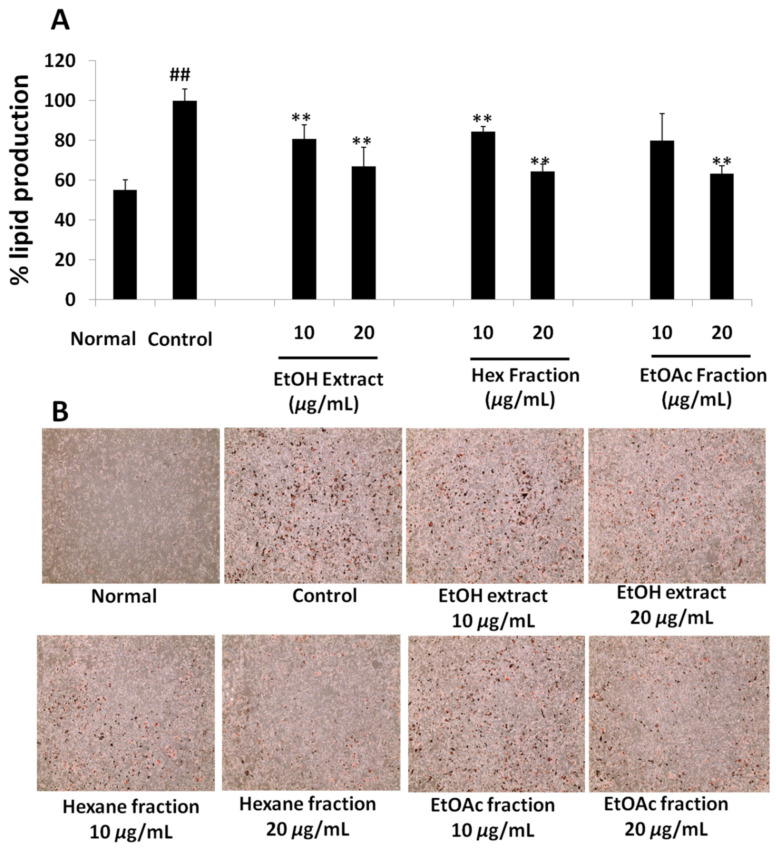
(**A**) Effect of ethanol extracts, hexane fraction and ethyl acetate fraction on percentage lipid deposition by 3T3-L1 cells using ORO assay. (**B**)Lipid accumulation in 3T3-L1 cell observed by EVOS XL microscope at 10× magnification after ORO staining. Each set of data represents mean of triplicate experiment ± standard deviation. Significant difference between the groups was calculated using two-tailed Student’s *t*-test. ** *p* ˂ 0.01 vs. control, ## *p* ˂ 0.01 vs. normal are used to represent significant difference in lipid production of the sample compared to non-treated control group.

**Figure 6:**
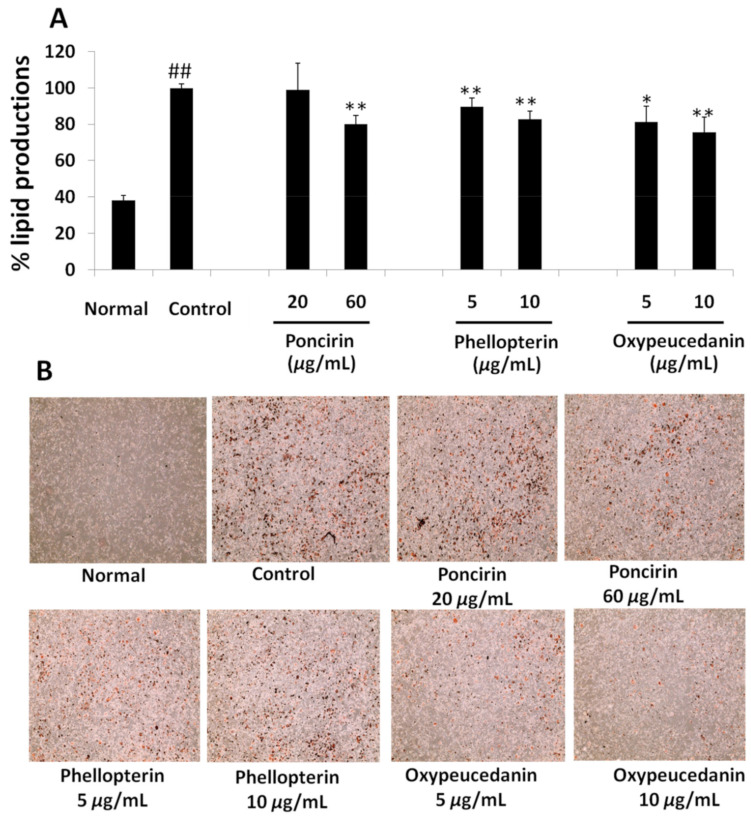
(**A**)Effect of poncirin, phellopterin and oxypeucedanin treatment on percentage lipid deposition by 3T3-L1 cells using ORO assay. (**B**) Lipid accumulation in 3T3-L1 cell observed by EVOS XL microscope at 10× magnification after ORO staining. Each set of data represents mean of triplicate experiments ± standard deviation. Significant difference between the groups was calculated using two-tailed Student’s *t*-test.* *p* ˂ 0.05 vs. control, ** *p* ˂ 0.01 vs. control, ## *p* ˂ 0.01 vs. normal are used to represent significant difference in lipid production of the sample compared to non-treated control group.

Anti-adipogenic activity was accessed by performing a quantification of lipids produced in the 3T3-L1 cell by ORO staining assay. The results in Figure 5 and Figure 6, Supplementary Figures S17 and S18 represent the mean production of lipids in non-differentiated and differentiated adipocytes with or without sample treatment. The photograph was taken after staining the cell with ORO to illustrate the intensity of the fat deposited in cells.

Result showed that the ethanol extract of fruit significantly inhibited the fat production in the 3T3-L1 cell line, as evidenced by the reduced deposition of cellular fat on the quantification by the ORO assay. The amount of fat produced was reduced to 80.97 ± 6.80% and 66.92 ± 9.7% at a dose of 10 µg/mL and 20 µg/mL of ethanol extract, respectively (Figure 5). This suggests that there must be active anti-adipogenic compounds in the extract, or a combination of constituents in the extract, collectively inhibiting adipocyte differentiation and fat deposition. Exactly the same pattern of lipid inhibition was observed in the treatment with the hexane fraction of the ethanol extract (Figure 5). In comparison to the control, 84.42 ± 2.55% and 64.37 ± 3.66% of lipid production were observed with the treatment of 10 µg/mL and 20 µg/mL of the hexane fraction, respectively (Figure 5). Similarly, a dose-dependent inhibition of lipid production was observed in ethyl acetate, butanol and water fractions. The ethyl acetate fraction at 20 µg/mL reduced the lipid production to 63.35 ± 3.98% (Figure 5), the butanol extract at 40 µg/mL reduced the lipid production to 83 ± 9.44% and the water fraction reduced the lipid production to 88.76 ± 4.24% (Figure S17), respectively. The reductions in fat deposition by these concentrations were statistically significant compared to the respective control groups. The cell morphology observed by the microscope showed no sign of toxicity in all of the treated groups (data not shown). The MDI media (differentiation media containing methylisobutylxanthine, dexamethasone and insulin)-treated control group showed a high deposition of white fat droplets in it, signifying adipogenesis induction in those cells. No such fat droplet deposition was observed in the normal fetal bovine serum (FBS)-treated control group after 8 days of the treatment period.

To determine the active constituents for the anti-adipogenic activity of Ponciri Fructus, seven compounds were isolated and separately treated to 3T3-L1 adipocytes in order to analyze whether they played a role in lipid inhibition. As shown in Figure 6, poncirin showed a significant reduction in lipid production to 80 ± 4.66% at a higher dose compared to the control. The naringin treatment showed a non-significant reduction in lipid production to 94.98 ± 11.84% at a 20 µ/mL concentration (Supplementary Figure S18). Auraptene, on the other hand, showed a significant increase in the production of lipids at a lower dose (5 µg/mL) to 109.46 ± 4.20%, but significantly decreased the production at the 10 µg/mL concentration to 85.86 ± 1.88% (Supplementary Figure S18). *β*-Sitosterol also increased the production of lipids at 10 µg/mL, but there was no significant change in the lipid production at a 20 µg/mL dose (Supplementary Figure S18). The production of lipids was increased by imperatorin at both doses (Supplementary Figure S18). Phellopterin reduced the production of lipids in a dose-dependent manner. At a 5 µg/mL concentration, the production of lipids was reduced to 89.76 ± 4.68%, whereas, at 10 µg/mL, the lipid production reached 82.94 ± 4.22%, which was significantly different compared to the control group (Figure 6). Lastly, oxypeucedanin showed a strong inhibition of the fat deposition at the end of the treatment period, as evidenced by fewer white fat droplets before the ORO stain (data not shown), and fewer ORO-stained cells after staining (Figure 6). A quantitative assay by dissolving the ORO stain in isopropanol showed that the inhibition of fat deposition by oxypeucedanin at both doses was significantly different compared to the control group. The inhibition of fat deposition was dose-dependent too. This compound reduced the fat production to 81.27 ± 8.69% at 5 µg/mL and 75.77 ± 8.11% at 10 µg/mL, respectively. This observation motivated us to further evaluate the molecular basis of the inhibition of adipogenesis by oxypeucedanin in the 3T3-L1 cell.

Overall, the ethanol extract, hexane fraction, ethyl acetate fraction, poncirin, phellopterin and oxypeucedanin showed a dose-dependent and significant inhibition of fat deposition in the 3T3-L1 cell, whereas the effects of other fractions and isolated compounds were non-significant.

#### 2.3.3. Regulation of Expression of Adipogenic Proteins by Oxypeucedanin

**Figure 7:**
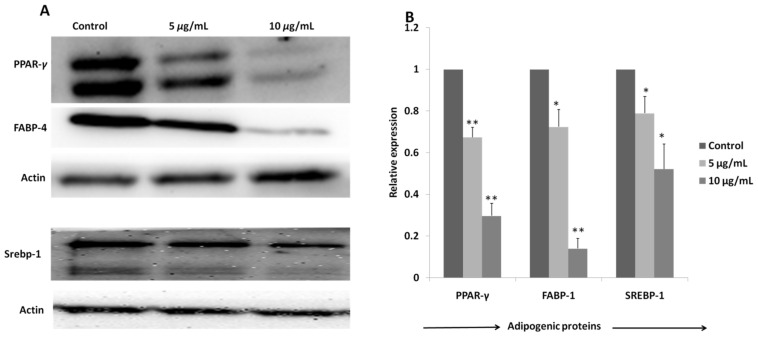
(**A**)Expression of PPAR-*γ*, FABP-4, SREBP-1 and actin proteins in 3T3-L1 cells on treatment with different doses of oxypeucedanin measured by Western blot analysis. (**B**) Densitometry analysis was performed to quantify protein levels and results were presented as mean of (*n* = 3) ± standard deviation. Significant difference between the groups was calculated using two-tailed Student’s *t*-test. * *p* ˂ 0.05 vs. control, ** *p* ˂ 0.01 vs. control are used to represent significant difference in expression of adipogenic proteins in treated group compared to non-treated control group.

Adipogenic marker proteins, PPAR-*γ*, FABP-4 and SREBP-1, were reduced dose-dependently in the treatment with oxypeucedanin, as evidenced by the band intensity of those proteins in the Western blot analysis (Figure 7A). A significant reduction in all of these adipogenic proteins was observed in the plot obtained by the quantification of the band intensity using Image J (Java1.8.0_172) software. The significant reduction in the protein expression strongly justified the least fat deposition in 3T3-L1 adipocytes observed in the oxypeucedanin-treated group during the ORO assay.

#### 2.3.4. Regulation of Expression of Adipogenic Gene by Oxypeucedanin

**Figure 8:**
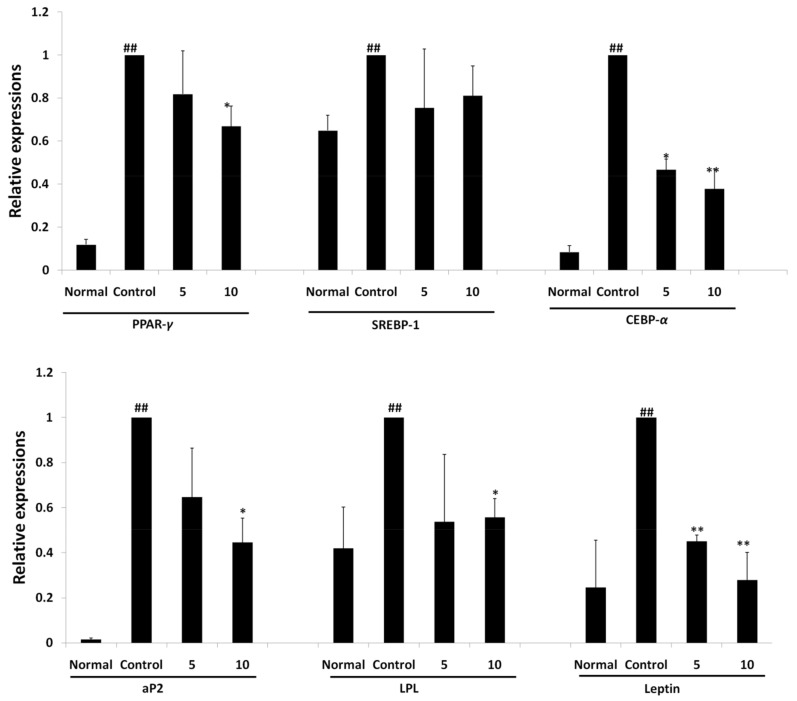
Relative expression of adipogenic genes PPAR-γ, SREBP-1, C/EBP-α, aP2, LPL and leptin in differentiated 3T3-L1 cells on treatment with oxypeucedanin at 5 µg/mL and 10 µg/mL concentrations. Results are expressed as mean ± standard deviation of triplicate experiment. Statistical significance difference was checked using two-tailed Student’s *t*-test. * *p* ˂ 0.05 vs. control, ** *p* ˂ 0.01 vs. control, ## *p* ˂ 0.01 vs. normal are used to represent significant difference in expression of gene between the groups.

As expression of proteins regulating adipogenesis was significantly reduced in the treatment of 3T3-L1 cells with oxypeucedanin, a further measurement of gene expression (transcription) was carried out by the RT-PCR technique. An evaluation of the expression of adipogenic genes was performed relative to the untreated MDI media control group. The results showed that the major adipogenic transcription factors PPAR-*γ*, SREBP-1, C/EBP-*α*, LPL, aP2 and leptin were significantly downregulated in a dose-dependent manner with oxypeucedanin (Figure 8), revealing its molecular mechanism of adipogenesis inhibition.

## 3. Discussion

Obesity is a serious global health concern that is raised these days. Due to the modern lifestyle, people are gaining weight more excessively than before. This increase in body weight due to a sedentary lifestyle is called passive obesity. By 2050, it is predicted that more than half of the world population could be obese [37]. This increased obesity does not come alone, but will also bring together several co-morbidities, such as type2 diabetes, cardiovascular disease, strokes, arthritis, various cancers and an associated healthcare cost. These facts necessitate global efforts to identify and develop ways to avoid or reduce the extent of obesity in the future [38].

A multi-faceted approach of lifestyle modification, dietary control, reduction in stress and regular exercise is recommended to control obesity [39,40]. Some anti-obesity therapeutic agents were also developed, but could not function well due to rebound obesity, psychological effects and toxicity associated with long-term use [41,42]. This scenario outlines how urgent it is to develop a safe and effective therapeutic agent for the management of obesity. Herbal-based therapies are considered to have minimal toxic responses compared to synthetic drugs [43]. Herbs that have shown good anti-adipogenic activity in scientific evaluation are *Piper nigrum* [44], *Camellia sinensis* [45], *Trigonella* *foenum-graecum* [46], *Zingiber* *officinale* [47], *Aegle marmelos* [48], *Sibiraea* *augustata* [49] and Corni Fructus [50], etc. Formulations obtained from herbal mixtures, such as Triphala [51], Vrikshamla [52], Dohongsamul-tang [53], Samchulgeonbi-tang [54], SH21B [55], Oyaksungi-san [56] and formulation F2 [57], etc. have also proven their efficacy for both the treatment of obesity and anti-adipogenic potential in in-vitro and in-vivo experiments. Anti-adipogenic compounds, such as platycodin D [58], *p*-synephrine [59], butein [60], viscothionin [61], adenanthin [62], idescarpin [63], rutin [64], asperuloside [65] and capsaicin [66], are also isolated and evaluated from herbal resources. In this study, Ponciri Fructus was evaluated for its anti-adipogenic activity in order to give scientific evidence toward having possible anti-obesity therapeutic effects.

Numerous scientific reports indicate the diverse biological activities of this medicinal fruit in cell and animal models. In addition, weight reduction was observed in high-fat-diet-fed mice models [20]. Still, the mechanism of weight reduction by Ponciri Fructus has not yet been understood. This has motivated us to study the anti-adipogenic effects of fruit extract, its fractions and its isolated compounds in the 3T3-L1 cell line. We have also identified the molecular mechanism involved in the anti-adipogenic effect of the active compound. The inhibition of the 3T3-L1 preadipocyte differentiation can be considered as a potential therapeutic target for obesity [67]. We first evaluated the antiadipogenic activity of the extract and fractions, followed by isolation of compounds from active fractions. We have altogether isolated seven compounds; among them, two were flavonoids, one was phytosteroid and four were coumarin derivatives.

Ethanol extract, hexane fractions and ethyl acetate fractions inhibited lipid deposition in differentiated 3T3-L1 cells more strongly compared to butanol and water fractions. Based on this observation, we decided to isolate the active compound from those fractions. The isolation of compounds from the hexane fraction gave us auraptene, *β*-sitosterol, imperatorin, phellopterin and oxypeucedanin. Auraptene increased the deposition of fat droplets in the 3T3-L1 cell at a lower dose, which coincides with the findings of Kuroyanagi et al. [68]. Auraptene acts as a PPARs agonist and enhances the production of adiponectin while reducing the monocyte chemoattractant protein, increasing the fat deposition in differentiated 3T3-L1 cells [68]. The proliferative effect of auraptene at a lower dose was also observed with an MTT assay (Figure 4). *β*-Sitosterol, a well-known functional food additive, and imperatorin, showed a significant increase in the lipid production at lower doses (Supplementary Figure S18). The increase in fat deposition by imperatorin might be due to an enhanced expression of PPAR-*γ* [69]. Phellopterin reduces fat deposition dose-dependently, and was significant compared to the control (Figure 6). This compound when administrated to the diabetic animal model reduced the blood level of glucose, triglyceride and cholesterol [69]. Oxypeucedanin is another isolated compound that inhibited the deposition of fat droplets in 3T3-L1 cells significantly at both doses (Figure 6). The inhibition of adipogenesis had not been reported for this compound before, but the hydroxylated derivative of oxypeucedanin showed a good binding affinity in PPAR-*γ* ligands [70]. In this study, we have evaluated the anti-adipogenic effects of oxypeucedanin for the first time. An evaluation of the mechanism of the action of this compound showed that it significantly reduced the expression of vital adipogenic proteins and genes.

PPAR-*γ* is a crucial adipocyte-specific binding protein that regulates adipogenesis. PPAR-*γ* expression promotes fibroblasts to convert into preadipocyte, which, again, differentiates into matured adipocyte by the feedback mechanism of C/EBP-*α*, SREBP-1 and FABP-4. FABP-4 further promotes the formation and storage of lipid droplets in differentiated adipose cells [4,71,72,73]. As shown in Figure 7, oxypeucedanin significantly inhibited the expression of these groups of proteins and transcription factors through inhibiting the process of adipogenesis.

Lipoprotein lipase (LPL) derived from adipocyte is one of the major regulators of adipogenesis, playing a major role in the efficient uptake and storage of fatty acid in cells. The higher the expression of the LPL gene, the more efficient the storage of fat in the adipocyte, resulting in hypertrophy of the cell [74]. Oxypeucedanin inhibited the expression of this gene through blocking the excessive storage of lipid droplets in the differentiated 3T3-L1 adipocyte (Figure 8).

Leptin is one of the adipokines responsible for regulating energy intake and expenditure. It is produced by adipocytes and conveys a paracrine signal to the hypothalamus by binding with a special hypothalamic receptor. This will control the feeding behavior of the animal, and the feedback response will arise using the JAK/STAT pathway. The activation of the leptin gene was observed during the process of adipogenesis. Therefore, an inhibition of leptin signaling could serve to control the process of adipogenesis [75,76]. The expression of leptin was significantly inhibited by the oxypeucedanin treatment (Figure 8) in 3T3-L1 adipocytes, indicating an inhibition of adipogenesis.

**Figure 9:**
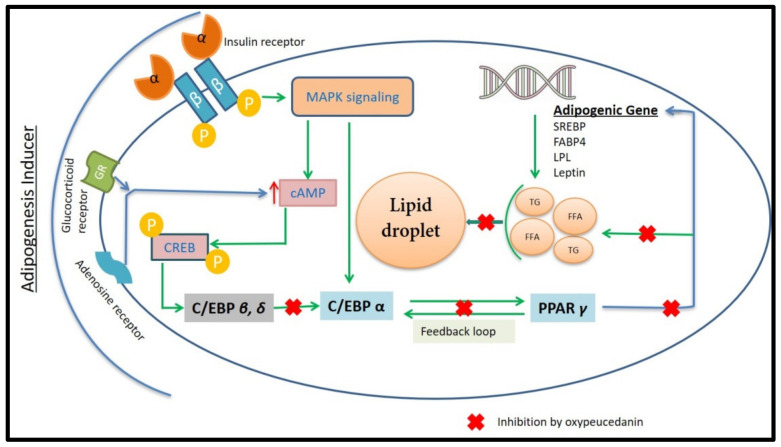
Possible inhibition pathway of oxypeucedanin in adipogenesis.

Adipocyte lipid binding protein (aP2) is another group of signaling molecules where their over-expression may lead to an increase in the accumulation of cholesterol and triglyceride as a result of increasing the expression of the scavenger receptor type AI responsible for lipid metabolism. The aP2 gene also links obesity to insulin resistance by regulating the expression of TNF-*α* [77,78]. The inhibition of this gene expression was observed in the treatment with oxypeucedanin, indicating its potential anti-adipogenic potential activity (Figure 8).A possible pathway for the inhibitory activity of oxypeucedanin in adipogenesis is presented in Figure 9.

## 4. Materials and Methods

### 4.1. Solvents, Chemicals and Instrumentation

All of the solvents used in the experiments were of (high performance liquid chromatography) HPLC grade. Hexane, dichloromethane, EtOAc, butanol, methanol, ethanol, isopropanol and acetonitrile were purchased from SK Chemical (Seongnam, Korea). Dimethyl sulphoxide (DMSO; Junsei Chemicals, Tokyo, Japan), DMSO-*d6* and chloroform (CDCl3; Cambridge Isotope Laboratories Inc., Tewksbury, MA, USA), silica gel (Kieselgel with mesh size 60, 70–230, and 230–400) and coated TLC plate (normal and reverse phase; Merck, Darmstadt, Germany), ODS YMC gel (YMC group, Kyoto, Japan), Sephadex^TM^ LH-20 (GE Healthcare, Danderyd, Sweden), Nucleodur 100-5 C18ec (Macherey-Nagel, Düren, Germany) and Halo RP-amide column (from Advanced Materials Technology, Wilmington, DE, USA) were used for the analysis of plant extract and quality/quantity determination of isolated compounds.

NMR data were recorded in JEOL Eclipse 500 FT-NMR spectrometer. LC-MS (from Water Corporation, Stockport, UK) equipped with ESI ionization, ultra-performance liquid chromatography (UPLC; Agilent Technologies, Santa Clara, CA, USA) equipped with binary pump, auto-sampler and photodiode array detector were used to identify the structure of isolated compounds.

### 4.2. Cell Culture and Bioassay Reagent

Mouse embryonic fibroblast (3T3-L1 preadipocyte) supplied by American type culture collection (ATCC) was used for anti-adipogenic assays. Dulbecco’s modified Eagle medium (DMEM), newborn calf serum (NCS) and fetal bovine serum (FBS) (Life Technologies Corporation, Waltham, MA, USA), 3-isobutyl-1methylxanthine (IBMX), dexamethasone (DXM), insulin, 10% formalin, isopropanol and Oil Red O (ORO; Sigma-Aldrich, Darmstadt, Germany), thiazolyl blue tetrazolium bromide (MTT; Alfa Aesar, Heysham, UK), lysis reagent (Quiagen Sciences, Germantown, MD, USA), Power Cyber Green and high capacity RNA-cDNA kit (Applied Biosystem, Warrington, UK), RIPA buffer (Thermo Scientific, Bedford, MA, USA), primary antibodies (Santa Cruz Biotechnology Inc., Dallas, TX, USA) and secondary antibodies (Millipore, Burlington, MA, USA) were used for cell assays.

### 4.3. Extraction, Fractionation and Isolation

**Figure 10:**
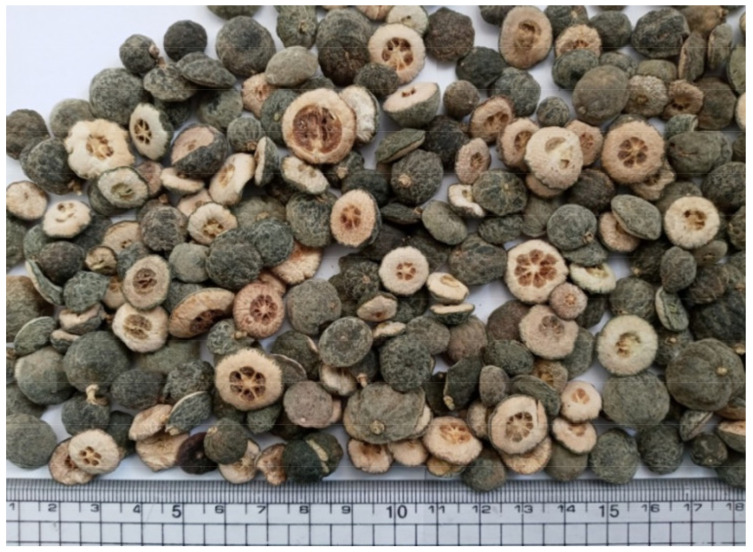
Photograph of Ponciri Fructus (dried immature fruits of *P. trifoliata*).

Dried immature fruit sample of *P. trifoliata* (Figure 10) was purchased from Humanherb, Korea (sample originated in China). Obtained fruit sample was stored at cool temperature until extraction.

Half-sliced immature fruit (8 kg) sample was refluxed with ethanol (96%) in 1:5 (*w*/*v*) fruits-to-solvent ratio at 85–90 °C for 2 h. The process was repeated three times and obtained liquid extract was filtered using filter paper (no. 20; Hyundai Micro, Seoul, Korea). Filtrate obtained was evaporated using rotatory evaporator to concentrate extract, and this was further lyophilized using freeze dryer. Completely dried extract was suspended in distilled water and fractionated successively using hexane, EtOAc and butanol to obtain respective fractions, and remaining suspension was obtained as water-soluble fraction. Each of the obtained fractions were evaporated and freeze dried and tested in 3T3-L1 cell line. Fractions showing potent bioactivity (hexane and EtOAc fractions) were further subjected for compound isolation using column chromatography.

Separation of EtOAc fraction (122 g) in silica column using dichloromethane:methanol:water (lower layer) in the ratio of 9:1.5:1 (1 L), 65:30:10 (4 L), 65:40:10 (4 L) afforded 20 (E1–E20) fractions based on TLC pattern. Fraction E4 was crystallized with methanol, filtered and freeze-dried to obtain 17.3 g of poncirin (**1**). Fraction E6 was further purified in ODS column using methanol:water in ratio 6:4 (1.5 L) and freeze-dried to obtain 2.083 g of naringin (**2**).

**Figure 11:**
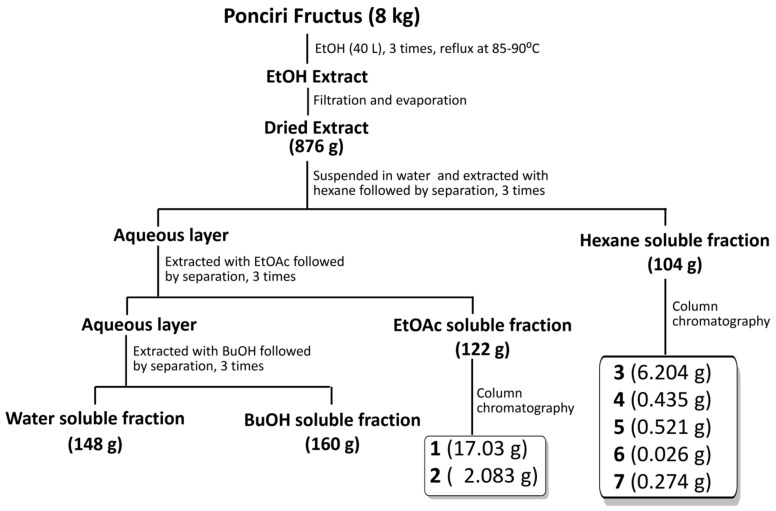
Flow chart for the fractionation and isolation of compounds from Ponciri Fructus.

Hexane fraction (104 g) separated in silica column using hexane and EtOAc mixture in the ratio 9:1 (6 L); 8:2 (6 L); 7:3 (6 L); 6:4 (4 L); 5:5 (4 L); 3:7 (2 L) afforded nineteen (H1–H19) fractions based on TLC patterns. Fraction H4 on isolation with ODS column using methanol:water in ratios of 8:2 (1 L); 9:1 (1 L); 10:1 (1 L) and isopropanol (0.5 L) afforded ten (H4-1–H4-10) total sub-fractions based on TLC pattern. Thus, obtained sub-fraction five (H4-5) on further purification in ODS column using acetonitrile:water in 9:1 (2 L) ratios afforded 6.204 g of auraptene (**3**). Sub-fraction eight from fraction four (H4-8) on purification using silica column with hexane and ethyl acetate mixture (8:2, 3 L) yielded 0.435 g of *β*-sitosterol (**4**). Fraction eight obtained from isolation of hexane fraction (H8) was isolated further in silica column using hexane and EtOAc mixture with ratio of 7:4 (3 L) to obtain a total of three sub-fractions (H8-1–H8-3). Sub-fraction one (H8-1) on further isolation in ODS column using acetonitrile and water mixture (8:2, 2 L) yielded imperatorin (**5**) and phellopterin (**6**). Obtained compounds were further purified in Sephadex LH20 using methanol as mobile phase. Fraction thirteen obtained from hexane fraction (H13) was further isolated with silica column using hexane and ethyl acetate (6:4, 3 L) mobile phage to obtain six sub-fractions (H13-1–H13-6), and sub-fraction four (H13-4) on further purification with ODS column using acetonitrile and water mixture (6:4, 2 L, thrice) afforded 274 mg of oxypeucedanin (**7**). The schematic representation of the extraction, fractionation and isolation is depicted in Figure 11 below.

### 4.4. Identification of Isolated Compounds

Obtained pure compounds were dissolved in DMSO-*d6* or CDCl_3_ solvents and were then evaluated for chemical structure using NMR spectroscopy. Acquired spectra were compared with reference spectra published in several journals to identify compound structure. Further IR spectra, LC-MS spectra and 2D NMR spectra were obtained to confirm predicted structure of compounds.

### 4.5. UPLC Instrumentation and Column Condition

Quantification of isolated compounds was carried out using UPLC from Agilent technologies (Santa Clara, CA, USA) equipped with binary pump, auto sampler and photodiode array detector. Halo 90 A, RP-Amide (2 µm × 2.1 mm × 150 mm) column from Advanced Materials Technologies was used to evaluate isolated compounds. Gradient elution system of water and acetonitrile used for optimum separation of peaks is given in Supplementary Table S1. Column temperature was adjusted to 35 °C, and injection volume for each sample was 2 µL. Standard calibration curve for poncirin, naringin, auraptene, imperatorin and oxypeucedanin was prepared by plotting different concentrations (1 mg/mL to 32.5 µg/mL using serial dilution) of standard compound in *x*-axis and peak area in *y*-axis. Evaluated compounds showed good linearity in concentration range injected, as evidenced by correlation coefficient (*R^2^*) ≥ 0.995. Thereafter, quantification of isolated compounds was carried out in ethanol extract and hexane fraction of fruit. Extract and hexane fraction were dissolved in methanol, and quantification of compounds was carried out using prepared standard calibration curves.

### 4.6. Sample Preparation for Cell Assay

Freeze-dried fruit extract, fractions and isolated compounds were dissolved in DMSO at concentration of 10 mg/mL stock concentration. The stock solutions were preserved in −20 °C until treatment in cell. The treatment concentrations were prepared by diluting stock solution with respective cell culture media.

### 4.7. Cell Assay on 3T3-L1 Pre-Adipocyte

#### 4.7.1. Cell Culture and Viability Assay

3T3-L1 preadipocyte (ATCC^®^ CL-173™, Manassas, VA, USA) was grown and sub-cultured in DMEM supplemented with 10% NCS and 1% penicillin–streptomycin antibiotics solution. The cultured cells were kept inside incubator, with humidified atmosphere at 37 °C and 5% CO2.

For cell viability assay, sub-cultured stock cells at around 70% confluency were collected and seeded into 48-well plate with cell density 2 × 10^3^ cells per well in DMEM supplemented with 10% NCS. The cells were then treated after 48 h with different concentrations of extract, fractions and isolated compounds in DMEM supplemented with 10% FBS and incubated for next 48 h. The viability was accessed using MTT assay kit following manufacturer protocol.

Cell viability was calculated as:

(1) $$\mathrm{Percentage}\;\mathrm{cell}\;\mathrm{viability}=\;\left(\mathrm{Sample}\;\mathrm{absorbance}/\;\mathrm{Control}\;\mathrm{absorbance}\right)\;\times \;100$$

#### 4.7.2. 3T3-L1 Preadipocyte Differentiation and Oil Red O Assay

Anti-adipogenic activity of samples was observed by measuring the amount of lipid deposited in differentiated 3T3-L1 adipocyte using Oil Red O (ORO) assay. Preadipocytes were seeded into 24-well plate at cell density of 5 × 10^3^ cells per well, and were left for confluency. Cell media were changed in 2-day intervals. After 2 days of observance of 100% confluency, cells were treated with different concentrations of sample in MDI differentiation media. The differentiation media were composed of IBMX-0.5 mM, dexamethasone (1 µM) and insulin (5 µg/mL) in DMEM supplemented with 10% FBS. Two days after treatment, differentiation media were replaced by adipocyte-maintaining media containing 5 µg/mL insulin in DMEM with 10% FBS. Then, on day 4 and day 6, the media were replaced with plain DMEM containing 10% FBS. Group with non-differentiated cell (normal group) grown in plain growth media (10% FBS) was used to monitor level of differentiation in treated cell. At the end (8th day) of treatment, cells were washed with phosphate-buffered saline (PBS) and fixed with formalin (10%). After 24 h, cells were viewed using EVOS XL (Life Technologies, Carlsbad, CA, USA) microscopes for morphology followed by ORO staining to determine lipid deposition in the cells. ORO-stained cells were again observed in microscope, followed by extraction of stain using 100% isopropanol for quantification of the deposited lipids. Optical absorbance was recorded at 520 nm using microplate reader, and percentage lipid deposition was calculated relative to percentage of control as follows:

(2) $$\mathrm{Percentage}\;\mathrm{lipid}\;\mathrm{deposition}=\;\left(\mathrm{Sample}\;\mathrm{absorbance}/\mathrm{Control}\;\mathrm{absorbance}\right)\;\times \;100$$

#### 4.7.3. RNA Extraction and Real Time PCR Analysis

Method described by Pandey et al. was used for total RNA extraction, cDNA synthesis and RT-PCR analysis [79]. Briefly, treated adipocytes at day 8 were washed with PBS and collected using QIAzol lysis reagent. RNA was extracted and 1 µg of mRNA was reverse transcribed using high capacity RNA-cDNA kit in thermal cycler. Gene expression was analyzed using real time quantitative PCR (RT-qPCR). cDNA was amplified using SYBER-GREEN PCR Master Mix in StepOnePlus RT-PCR system. The 2-ΔCT method was used to calculate relative mRNA expression using mouse *β*-actin as a reference gene.

#### 4.7.4. Protein Extraction and Western Blotting

Cells were treated with different concentrations of sample and harvested using ice-cold RIPA buffer using cell scrapper. After vertexing for 30 min, the sample was centrifuged at 14,000 rpm for 20 min at 4 °C, and the supernatant was collected. BCA protein assay kit was used to quantify protein concentration. Protein of around 30 µg was loaded and separated in SDS-polyacrylamide gel electrophoresis. Gels were transferred to nitrocellulose membrane at 150 mA for 1 h and blocked with 5% skim milk and 0.1% Tween20 for 2 h at room temperature. Blots were incubated with primary antibodies overnight at 4 °C followed by secondary antibodies for 1 h. Protein bands were determined by gel image system using chemo-illuminance. The obtained protein bands were photographed and analyzed using ImageJ software (Java1.8.0_172).

### 4.8. Statistical Analysis

Data are presented as mean of triplicate experiments ± standard deviation. Statistical significance between the groups was calculated using two-tailed Student’s *t*-test in Microsoft Excel 2010. A 95% confidence, as represented by *p* ˂ 0.05, was considered as statistically significant difference between the groups.

## 5. Conclusions

Based on all of the observations, we came to the conclusion that the ethanol extract, hexane fraction and ethyl acetate extract of Ponciri Fructus inhibited lipid deposition in differentiated 3T3-L1 cells. Isolated poncirin and auraptene showed anti-adipogenic activity at a higher dose, whereas, on the other hand, auraptene and imperatorin increased the lipid deposition at lower doses. Oxypeucedanin and phellopterin inhibited the lipid deposition in the 3T3-L1 cell dose-dependently, as observed by the ORO assay. Oxypeucedanin showed this activity by inhibiting the expression of adipogenic markers, such as PPAR-*γ*, SREBP-1, C/EBP-*α*, FABP-4, LPL, aP2 and leptin. Further animal experiments are needed to identify how this bioactive coumarin will act on animal and human subjects.

## Figures and Tables

**Table 1:** Quantification of isolated compounds using UPLC.

Standard Compounds	Regression Equation	R2	Content (µg/mg)	Quantified in
Poncirin (**1**)	y = 12.594x + 287.77	0.999	166.21 ± 0.95	Ethanol extract
Naringin (**2**)	y = 15.663x − 149.67	0.998	78.78 ±1.41	
Auraptene (**3**)	y = 18.477x + 6.3058	0.999	12.57 ± 0.06	
Imperatorin (**5**)	y = 13.476x + 674.38	0.996	35.63 ± 0.27	Hexane fraction
Oxypeucedanin (**7**)	y = 16.141x + 179.16	0.995	23.10 ± 0.18	

## Data Availability

The datasets used and/or analyzed during this study are available from the corresponding author on reasonable request.

## Supplementary Materials

Figure S1: UPLC overlay chromatogram of poncirin (**1**) isolated from fruit of *P. trifoliata* (A) and standard poncirin (B) at 280nm, together with UV spectra of poncirin, Figure S2: UPLC chromatogram of naringin (**2**) isolated from fruit of *P. trifoliata* (A) and standard naringin (B) at 280nm, together with UV spectra of naringin, Figure S3:^13^C (A) and ^1^H (B) NMR spectra of poncirin (**1**), Figure S4: ^13^C (A) and ^1^H (B) NMR spectra of naringin (**2**), Figure S5:^13^C (A) and ^1^H (B) NMR spectra of auraptene (**3**), Figure S6: UV spectra (A) and LCMS spectra (B) of auraptene (**3**), Figure S7: ^13^C (A) and ^1^H (B) NMR spectra of *β*-Sitosterol (**4**), Figure S8: ^13^C (A) and ^1^H (B) NMR spectra of imperatorin (**5**), Figure S9: IR spectra of imperatorin (**5**), Figure S10: LCMS spectra of imperatorin (**5**), Figure S11: ^13^C (A) and 1H (B) NMR spectra of phellopterin (**6**), Figure S12: IR spectra of phellopterin (**6**), Figure S13: ^13^C (A) and ^1^H (B) NMR spectra of oxypeucedanin (**7**), Figure S14: DEPT NMR spectra of oxypeucedanin (**7**), Figure S15: IR spectra of oxypeucedanin (**7**), Figure S16: UV spectra of oxypeucedanin (**7**), Figure S17: Effect of butanol fraction and water fraction on lipid deposition by 3T3-L1 cells using ORO assay, Figure S18: Effect of butanol fraction (A), water fraction (B), naringin (C), auraptene (D), *β*-sitosterol (E) and imperatorin (F) on lipid deposition by 3T3-L1 cells using ORO assay, Table S1: Solvent condition of UPLC analysis.

## Sample Availability

Samples of the compounds poncirin, naringin, auraptene, *β*-sitosterol, imperatorin and oxypeucedanin are available from the authors.
