# Supplementary material for: Anti-Obesity Potential of Ponciri Fructus: Effects of Extracts, Fractions and Compounds on Adipogenesis in 3T3-L1 Preadipocytes
Source: Molecules. 2022 Jan 20;27(3):676. doi: 10.3390/molecules27030676 (PMC8839251; doi:10.3390/molecules27030676)
Supplement: Supplementary file 1 [file molecules-27-00676-s001.zip › molecules-1529231 supplementary final to conversion.pdf]

molecules

MDPI

Supplementary Material

Anti-Obesity Potential of Ponciri Fructus: Effects of Extracts,Fractions and Compounds on Adipogenesis in 3T3-L1 Preadipocytes

Gopal Lamichhane [1], Prakash Raj Pandeya [1], RamakantaLamichhane [1], Su-jin Rhee[2], Hari Prasad Devkota [3] and Hyun-Ju Jung [1,*]

[1] Department of Oriental Pharmacy and Wonkwang-Oriental Medicines Research Institute, Wonkwang University, Iksan 570-749, Korea; lamichhanegopal1@gmail.com (G.L.); pandeya.praj@gmail.com (P.R.P.); clickrama@hotmail.com (R.L.)
[2] Department of Pharmacy, College of Pharmacy, Wonkwang University, Iksan 570-749, Korea; rheesj05@wku.ac.kr
[3] Graduate School of Pharmaceutical Sciences, Kumamoto University, 5-1 Oe-honmachi, Chuo ku, Kumamoto 862-0973, Japan; devkotah@kumamoto-u.ac.jp
* Correspondence: hyun104@wku.ac.kr; Tel.: +82-63-850-6814

Table S1. Solvent condition of UPLC analysis.

Time	Acetonitrile (%)	Water (%)	FLOW (mL/min)
0.00	0	100	0.2
5.00	60	40	0.2
10.00	100	0	0.2
15.00	100	0	0.2

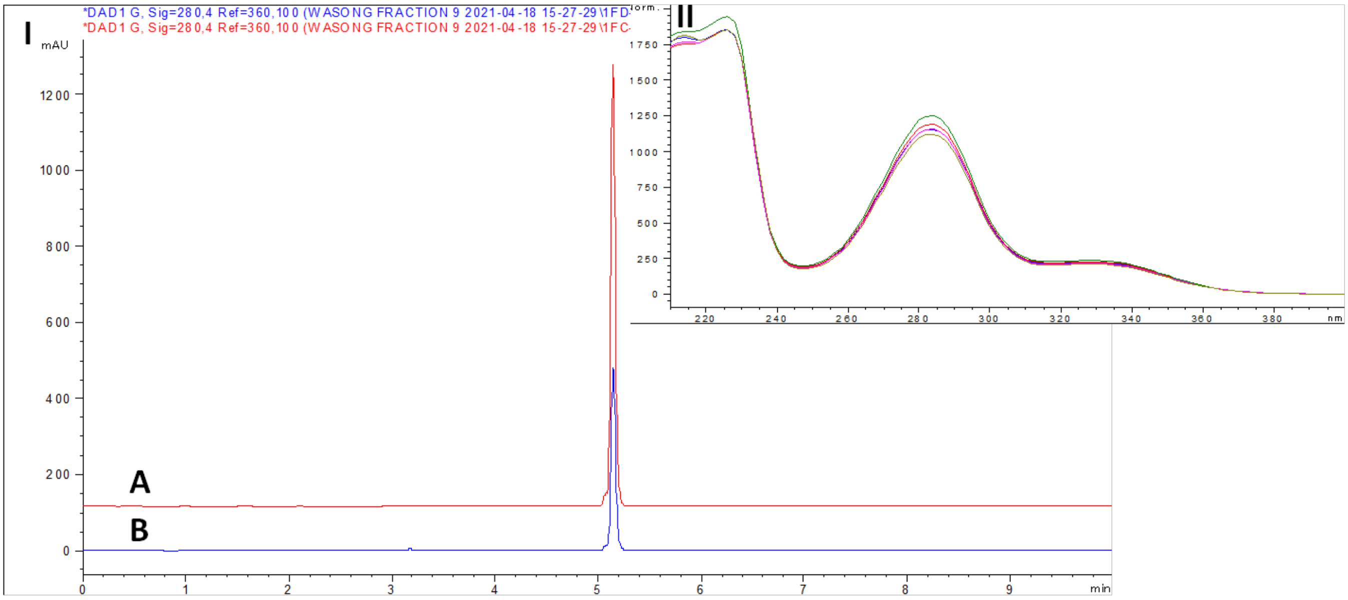

Figure S1. (I) UPLC overlay chromatogram of Poncirin(**1**) isolated from fruit of *P. trifoliata* (A) and standard poncirin (B) at 280nm together with (**II**) UV spectra of Poncirin.

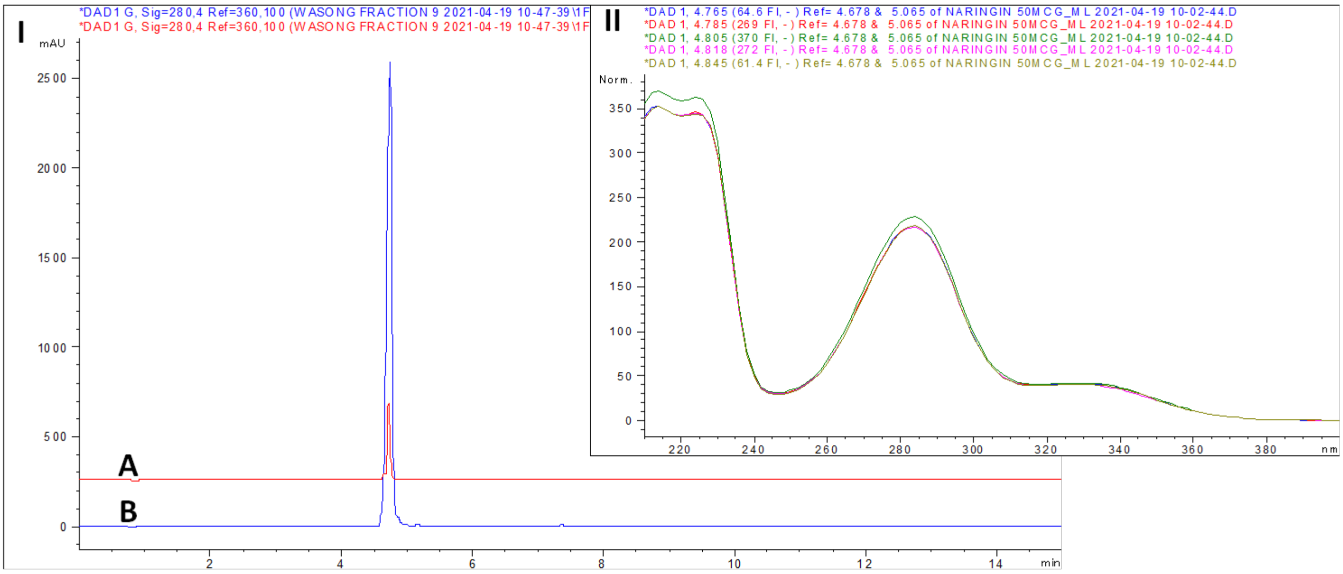

Figure S2. **(I)**UPLC chromatogram of Naringin (**2**) isolated from fruit of *P. trifoliata* (A) and standard naringin (B) at 280nm together with (**II**) UV spectra of Naringin.

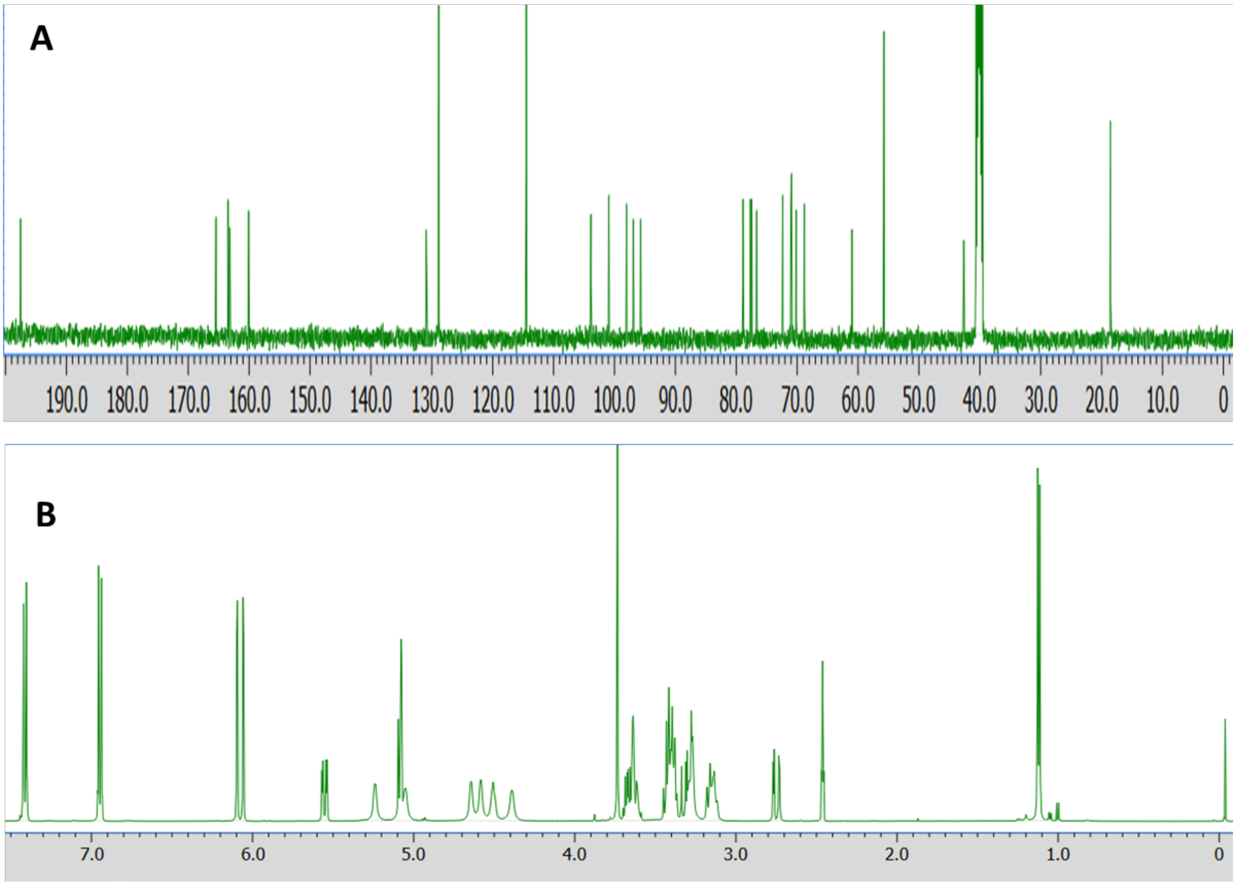

Figure S3. ^{13}C- (**A**) and ^{1}H-NMR (**B**) spectra Poncirin (**1**) in DMSO-*d*$_6$.

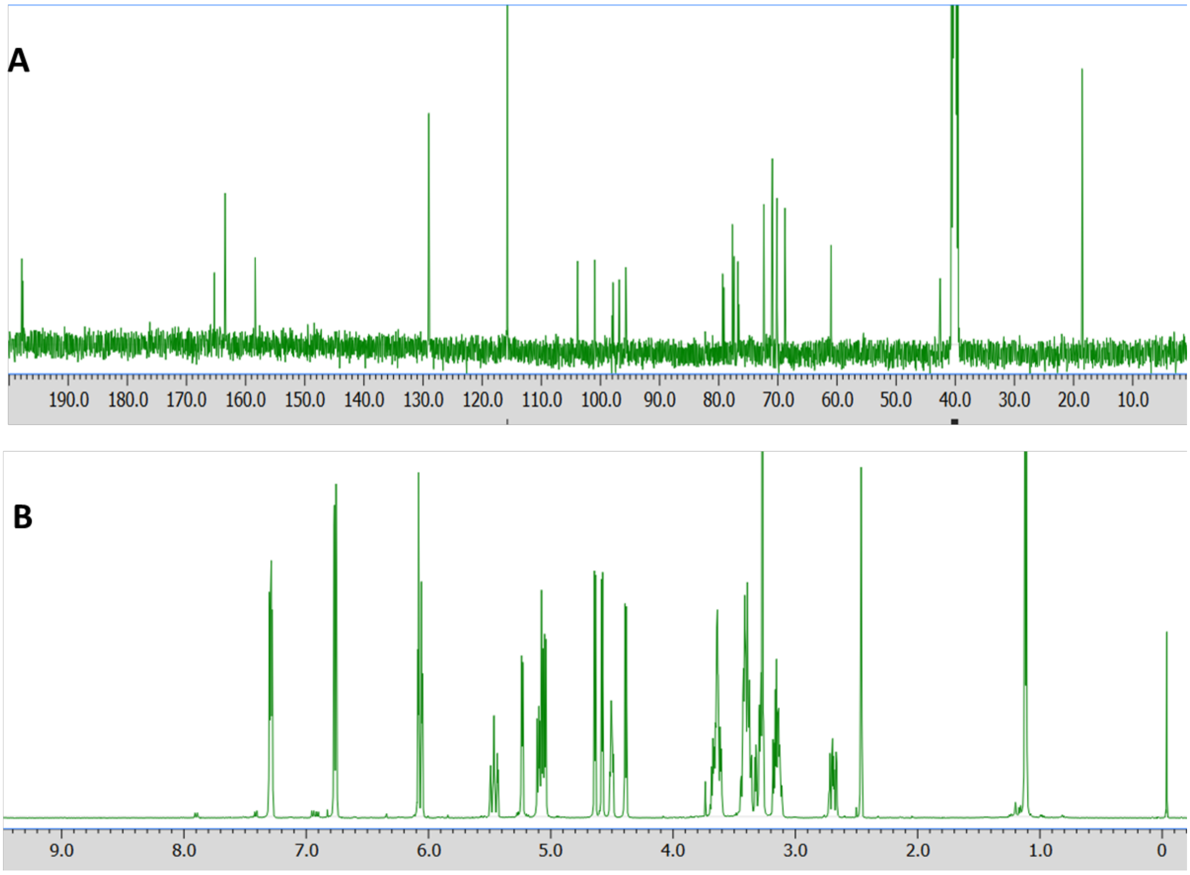

Figure S4. ^{13}C- (**A**) and ^{1}H-NMR (**B**) spectra of Naringin (**2**) in DMSO-d_6.

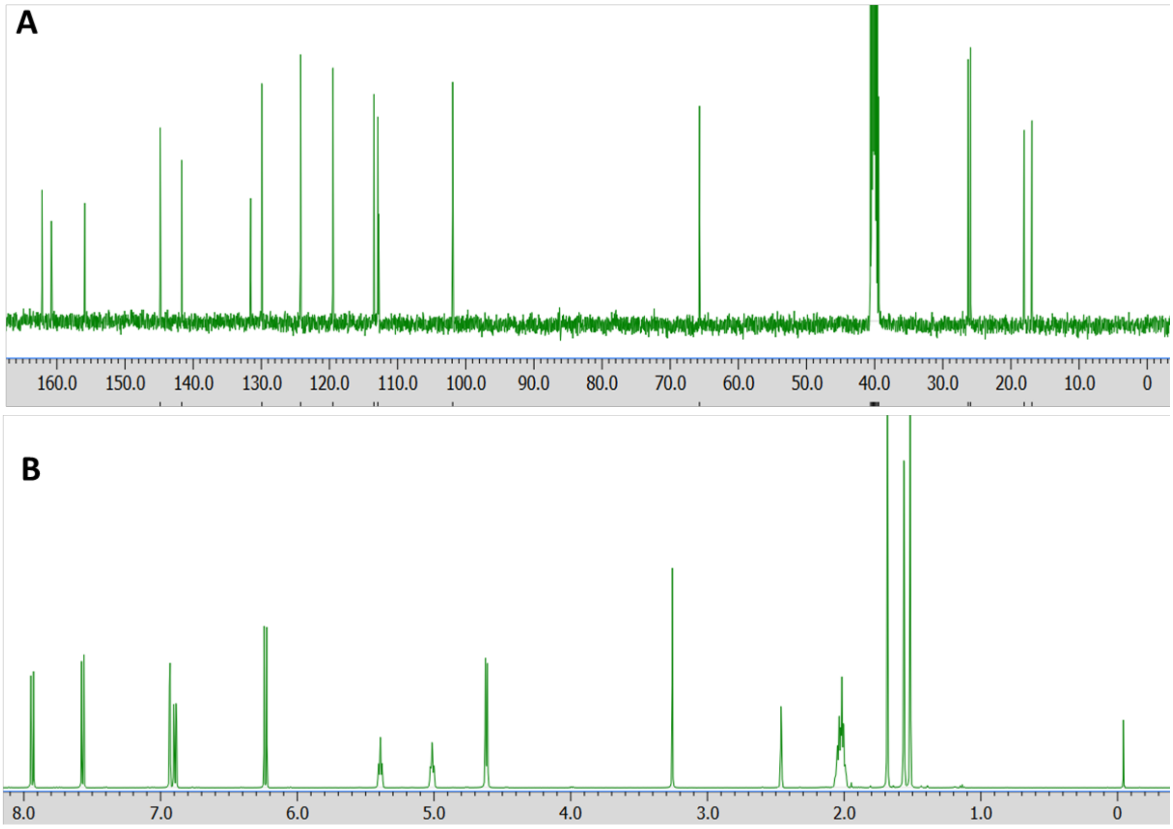

Figure S5. ^{13}C- (**A**) and ^{1}H-NMR(**B**) spectra of Auraptene (**3**) in DMSO-d_6.

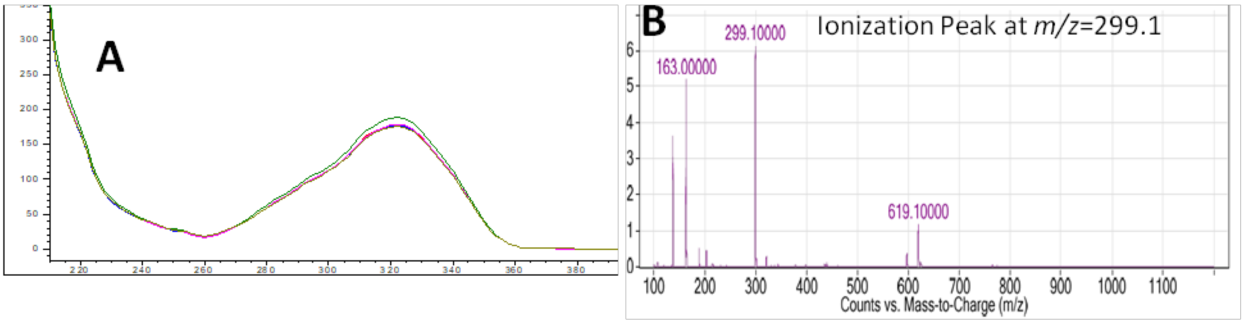

Figure S6. UV spectra (**A**) and LCMS spectra (**B**) of Auraptene (**3**).

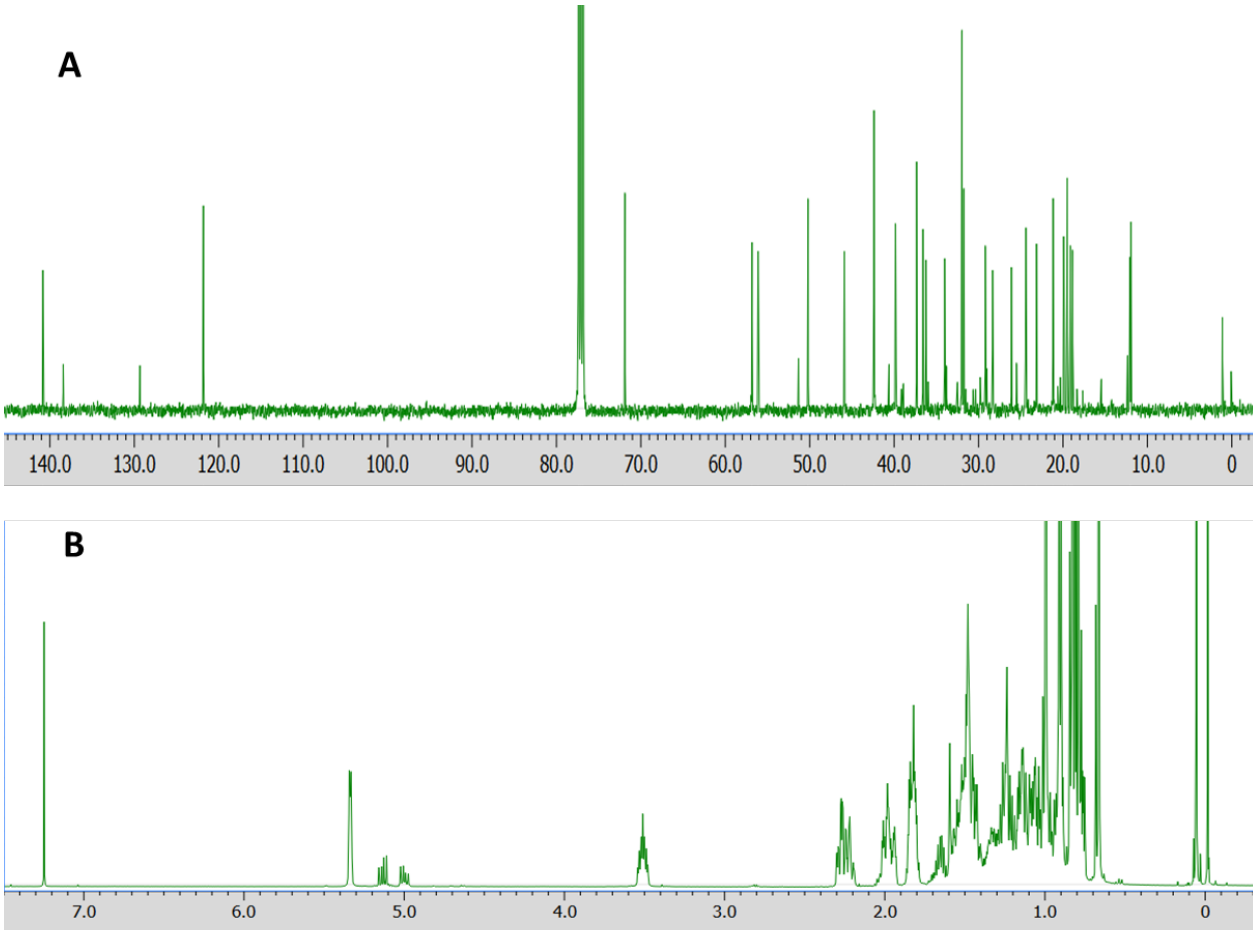

Figure S7. ^{13}C (**A**) and ^{1}H (**B**) NMR spectra of *β*-Sitosterol (**4**) in $CHCl_3$.

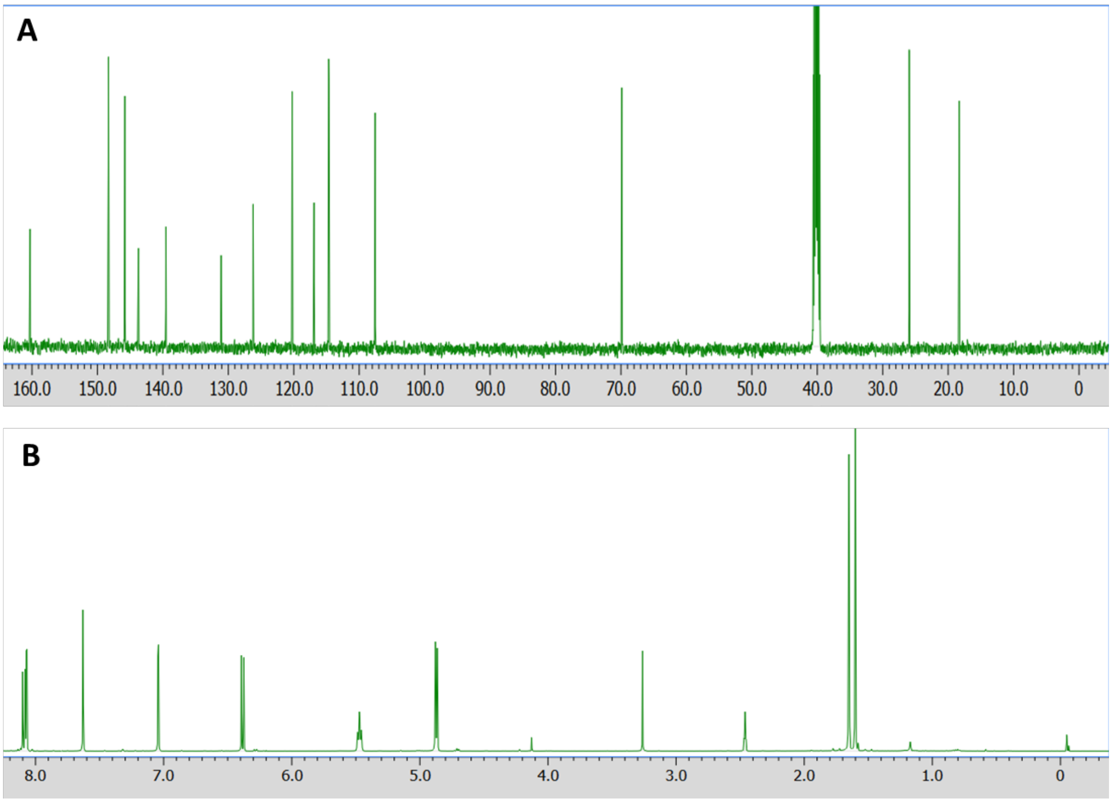

Figure S8. ^{13}C (**A**) and ^{1}H (**B**) NMR spectra of Imperatorin (**5**) in DMSO-d_6.

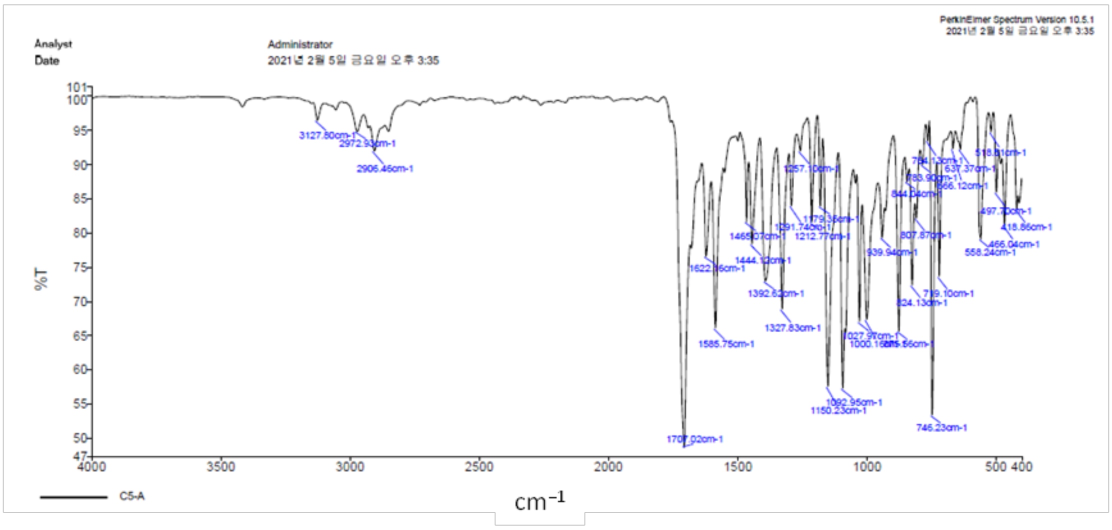

Figure S9. IR spectra of Imperatorin (**5**).

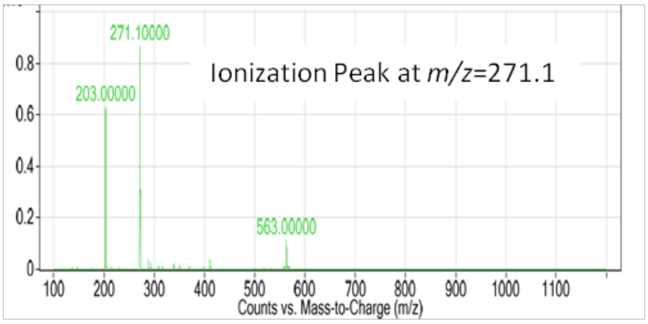

Figure S10. LCMS spectra of Imperatorin (**5**).

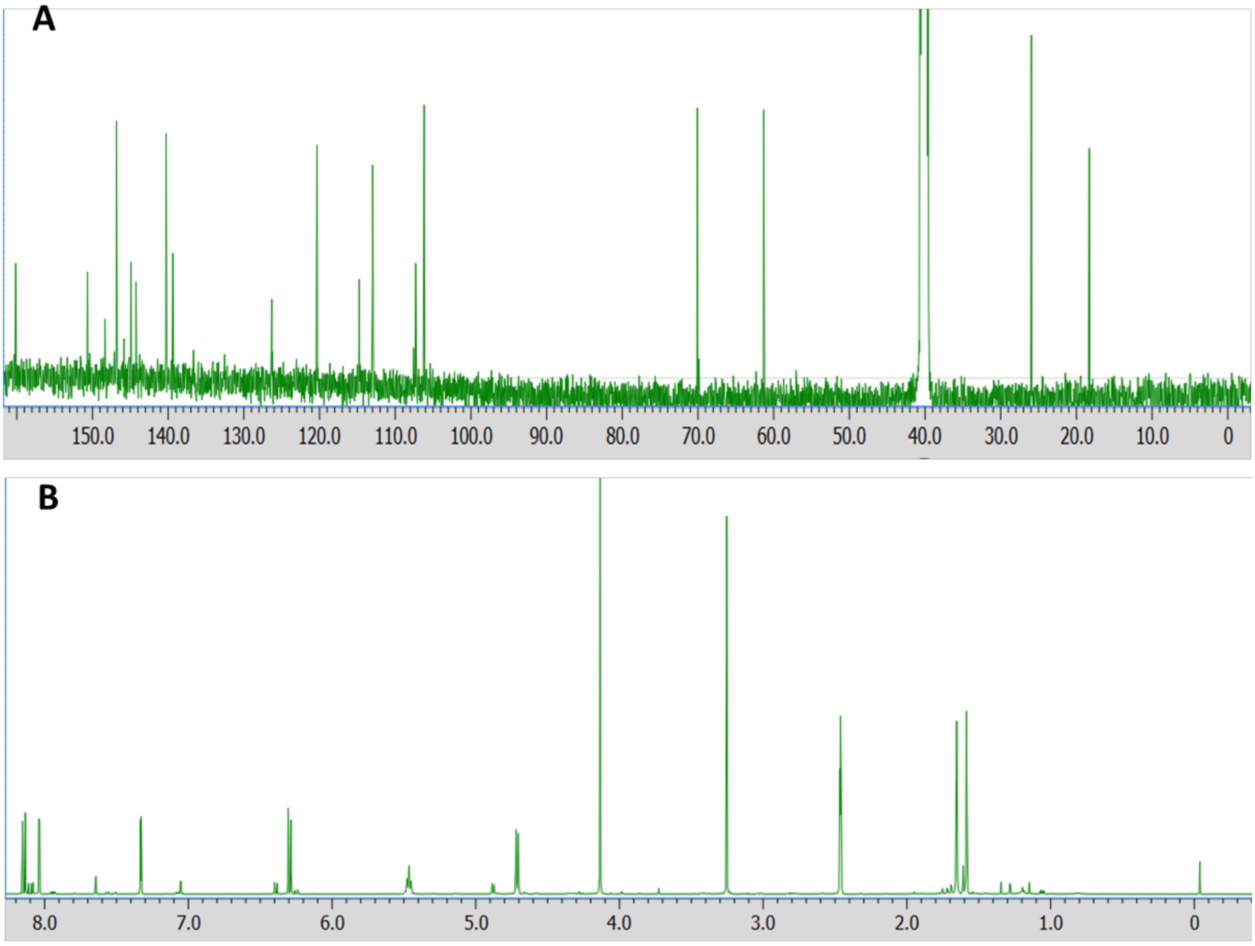

Figure S11. ^{13}C (**A**) and ^{1}H (**B**) NMR spectra of Phellopterin (**6**) in DMSO-d_6.

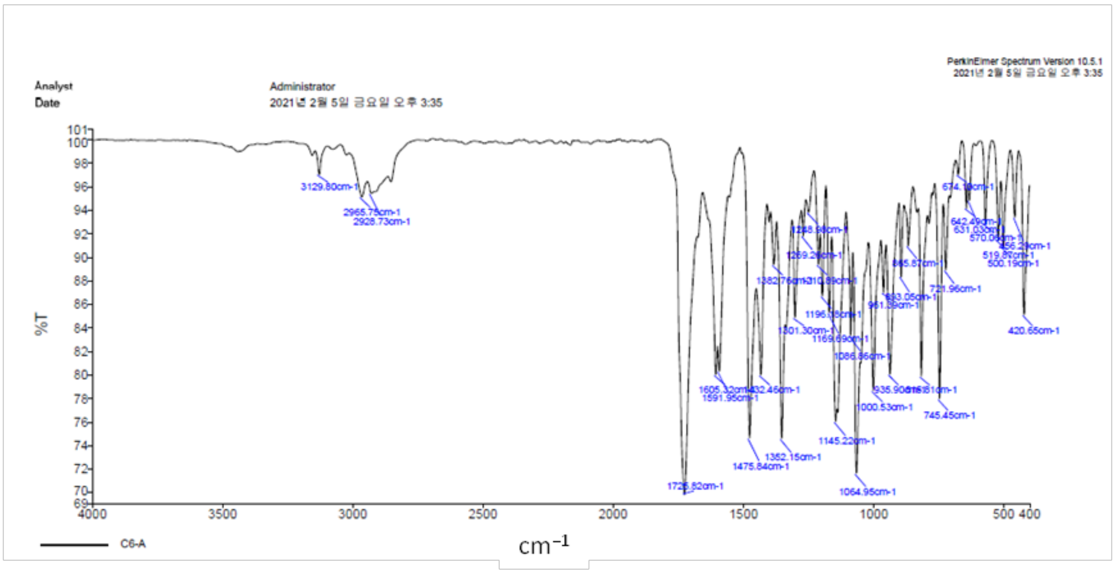

Figure S12. IR spectra of Phellopterin (**6**).

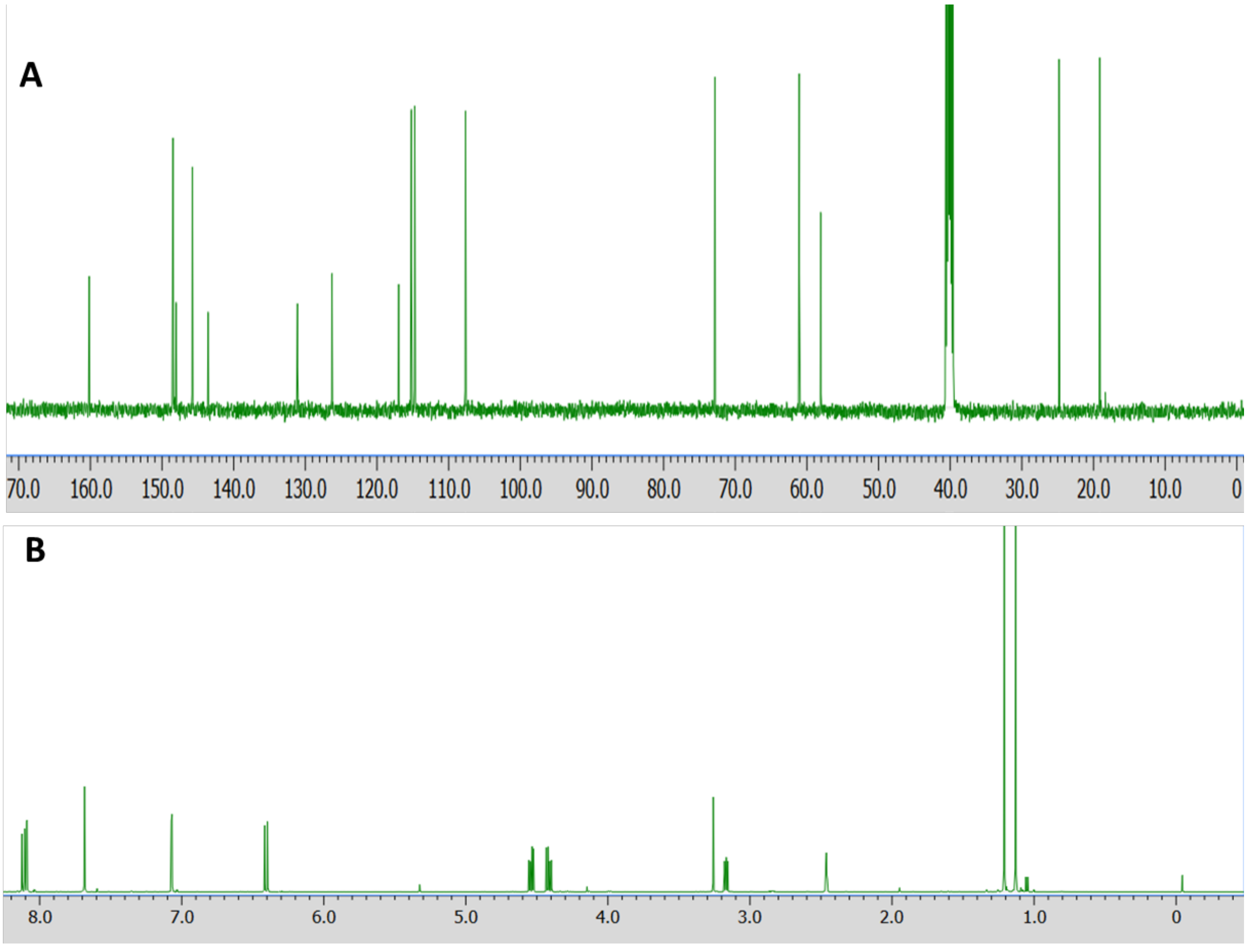

Figure S13. ^{13}C (**A**) and ^{1}H (**B**) NMR spectra of Oxypeucedanin (**7**) in DMSO-d_6.

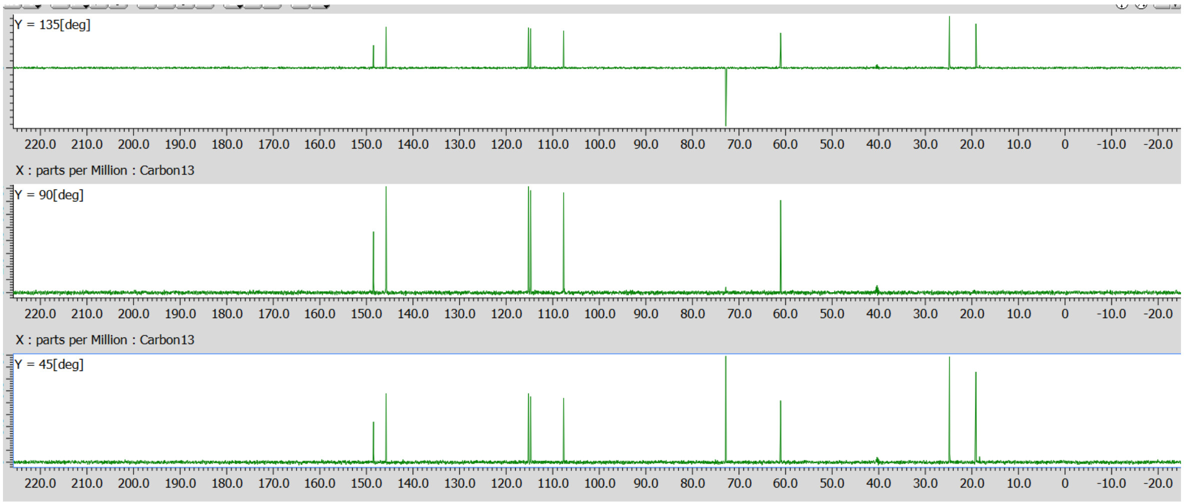

Figure S14. DEPT NMR spectra of Oxypeucedanin (**7**) in DMSO-*d*6.

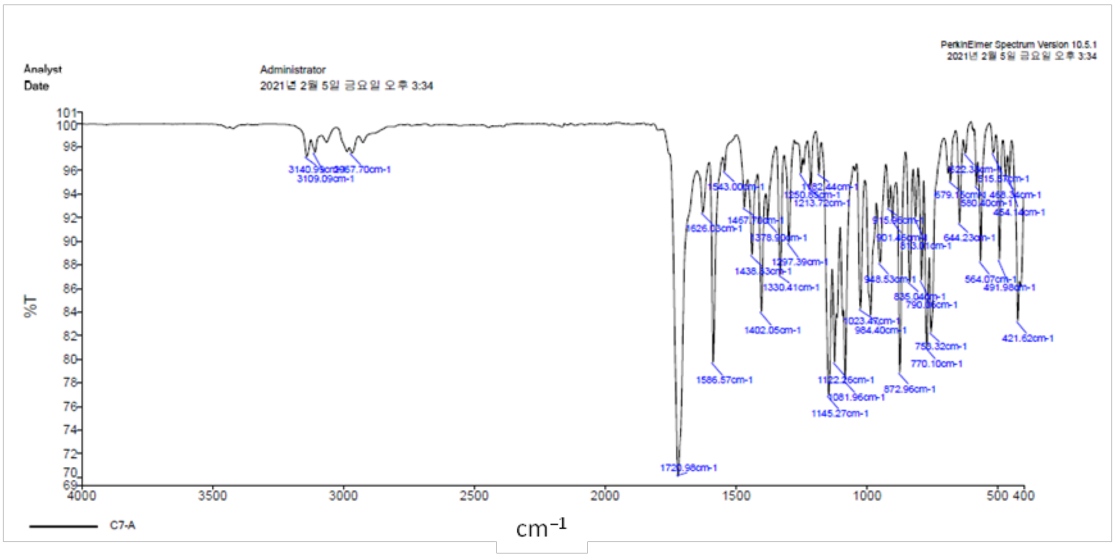

Figure S15. IR spectra of Oxypeucedanin (**7**).

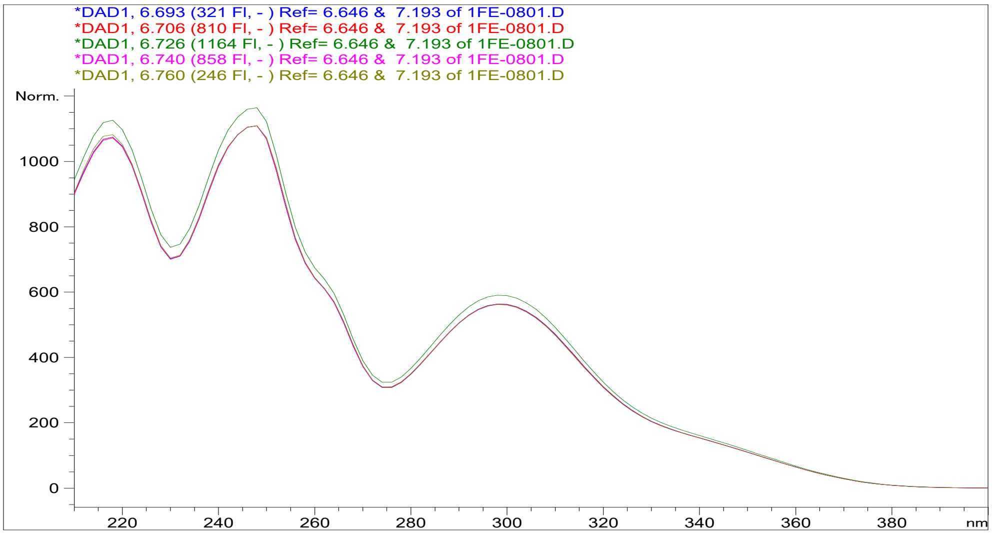

Figure S16. UV spectra of Oxypeucedanin (**7**).

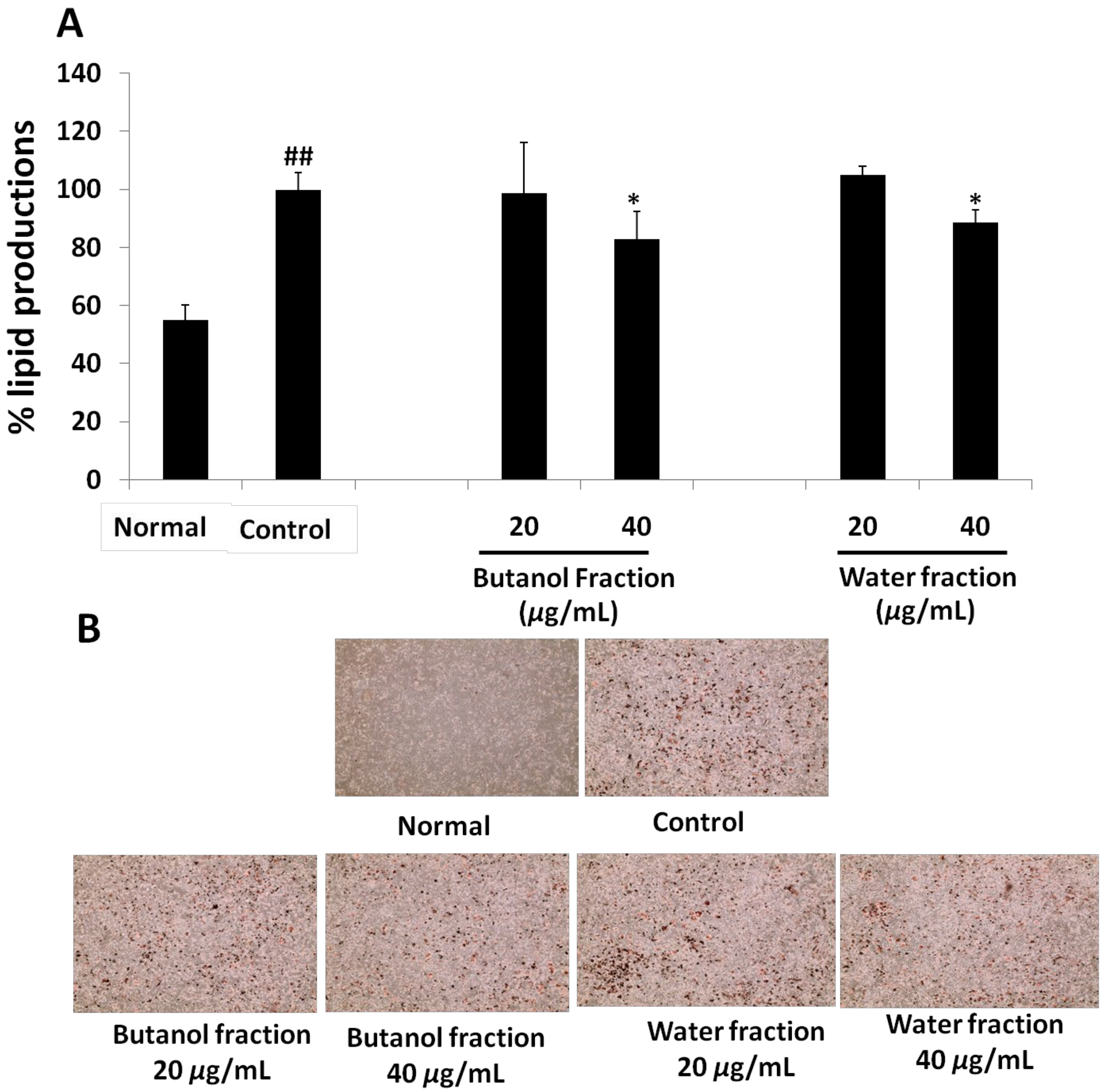

Figure S17. (**A**) Effect of butanol fraction and water fraction on percentage lipid deposition by 3T3-L1 cells using ORO assay. (**B**) Lipid accumulation in 3T3-L1 cell observed by EVOS XL microscope at 10× magnification after ORO staining. Each data represents mean of triplicate experiment ± Standard deviation. Significant difference between the groups was calculated using two tailed student *t*-test. *$p<0.05$ vs control, ## $p<0.01$ vs normal is used to represent significant difference in lipid production between the group.

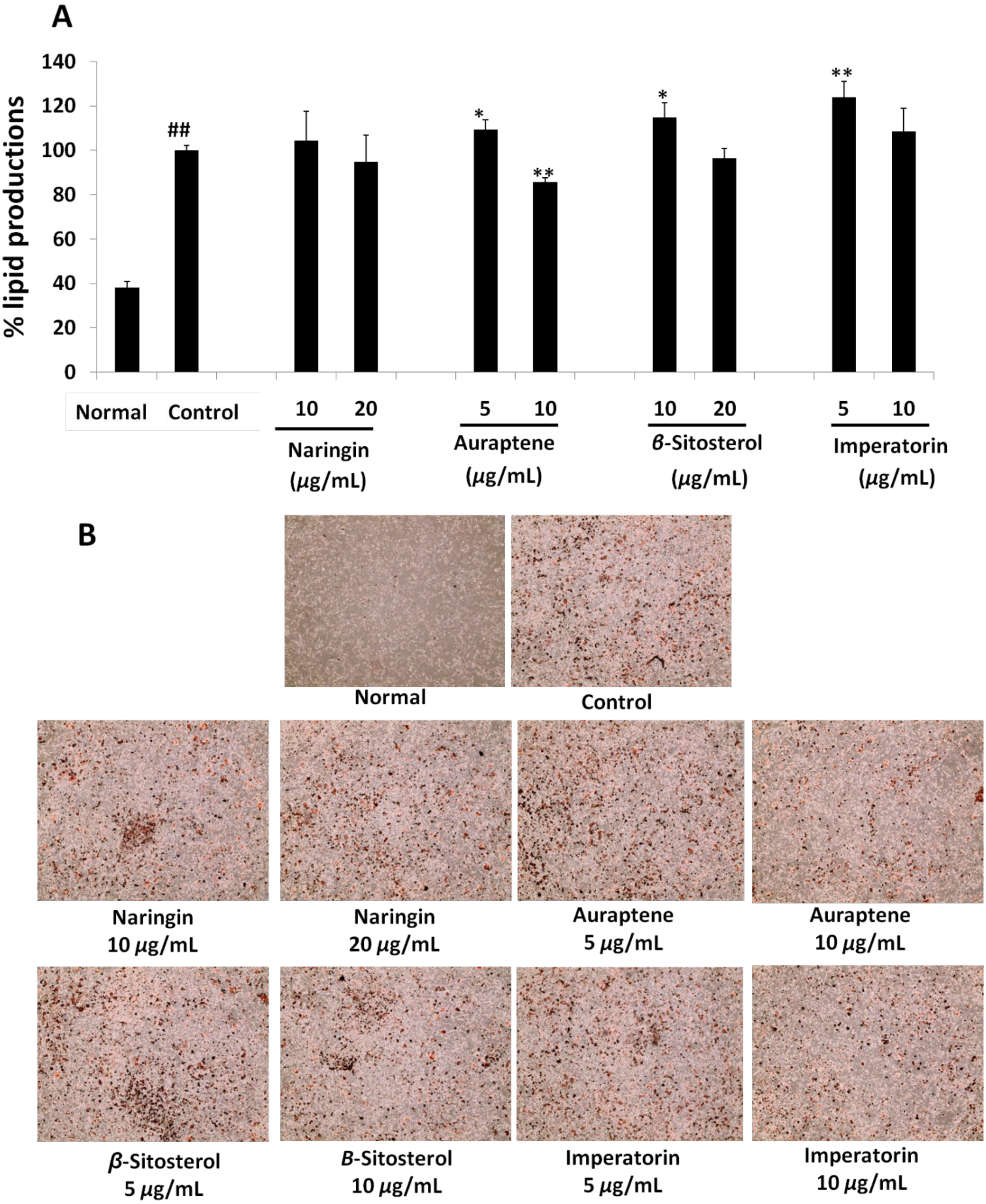

Figure S18. (**A**) Effect of butanol fraction, water fraction, Naringin, Auraptene, *β*-sitosterol and Imperatorin on percentage lipid deposition by 3T3-L1 cells using ORO assay. (**B**) Lipid accumulation in 3T3-L1 cell observed by EVOS XL microscope at 10× magnification after ORO staining. Each data represents mean of triplicate experiment ± Standard deviation. Significant difference between the groups was calculated using two tailed student t-test. *p<0.05 vs control, **p<0.01 vs control, ## p<0.01 vs normal is used to represent significant difference in https://doi.org/10.1186/s12906-018-2423-2. https://doi.org/.
